# Determining consistent prognostic biomarkers of overall survival and vascular invasion in hepatocellular carcinoma

**DOI:** 10.1098/rsos.181006

**Published:** 2018-12-05

**Authors:** Otília Menyhárt, Ádám Nagy, Balázs Győrffy

**Affiliations:** 1 2nd Department of Pediatrics, Semmelweis University, H-1094 Budapest, Hungary; 2 MTA TTK Lendület Cancer Biomarker Research Group, Institute of Enzymology, Hungarian Academy of Sciences, Magyar tudósok körútja 2, H-1117 Budapest, Hungary

**Keywords:** hepatocellular carcinoma, liver cancer, survival, biomarker, vascular invasion

## Abstract

*Background:* Potential prognostic biomarker candidates for hepatocellular carcinoma (HCC) are abundant, but their generalizability is unexplored. We cross-validated markers of overall survival (OS) and vascular invasion in independent datasets. *Methods:* The literature search yielded 318 genes related to survival and 52 related to vascular invasion. Validation was performed in three datasets (RNA-seq, *n* = 371; Affymetrix arrays, *n* = 91; Illumina gene chips, *n* = 135) by uni- and multivariate Cox regression and Mann–Whitney *U*-test, separately for Asian and Caucasian patients. *Results:* One hundred and eighty biomarkers remained significant in Asian and 128 in Caucasian subjects at *p* < 0.05. After multiple testing correction *BIRC5* (*p* = 1.9 × 10^−10^), *CDC20* (*p* = 2.5 × 10^−9^) and *PLK1* (*p* = 3 × 10^−9^) endured as best performing genes in Asian patients; however, none remained significant in the Caucasian cohort. In a multivariate analysis, significance was reached by stage (*p* = 0.0018) and expression of *CENPH* (*p* = 0.0038) and *CDK4* (*p* = 0.038). *KIF18A* was the only gene predicting vascular invasion in the Affymetrix and Illumina cohorts (*p* = 0.003 and *p* = 0.025, respectively). *Conclusion:* Overall, about half of biomarker candidates failed to retain prognostic value and none were better than stage predicting OS. Impact: Our results help to eliminate biomarkers with limited capability to predict OS and/or vascular invasion.

## 1. Background

In spite of tremendous efforts toward the discovery of novel prognostic or predictive biomarkers in solid tumours, less than 1% of these are estimated to enter clinical practice [1]. One critical component behind the high failure rate is poor reporting of key study elements hampering the interpretability and clinical applicability of prognostic studies [2]. Another critical requirement would be the validation of the findings by independent investigators in independent datasets, as promising biomarkers should provide reproducible results when tested in external samples.

Worldwide, liver cancer (LC) is the second leading cause of cancer-related mortality, with the vast majority of cases (83%) occurring in the less developed parts of the world causing a major health crisis in Eastern and Southeastern Asia [3]. Nonetheless, LC is also on the rise in the European Union (47 000 deaths per year) with the highest incidence in Southern Europe [4], and despite decreasing death rates for all cancers combined, LC burden is growing rapidly in the USA as well [5].

Hepatocellular carcinoma (HCC) accounts for up to 90% of primary liver malignancies with a highly unfavourable prognosis due to fast growth, early hepatic metastasis and rapid multidrug resistance. Chronic liver diseases linked to hepatitis infections, diabetes mellitus and fatty liver disease are frequently in the background of HCC [6]. Disease aetiology differs across races: in Asia, endemic for HBV and HCV, most HCCs develop as a consequence of chronic viral infection. In developed countries, HCC is linked primarily to chronic liver disease caused by cirrhosis from excessive alcohol consumption, diabetes mellitus or non-alcoholic fatty liver disease [6]. As a result of multiple causal factors, HCC is one of the most heterogeneous cancers with a highly variable clinical course. Abbreviations are listed in table 1.

**Table 1.** List of Abbreviations.

AFP	alpha-fetoprotein
CCKR	gastrin and cholecystokinin receptors mediated signalling network
CIN	chromosome instability
DAVID	Database for Annotation, Visualization and Integrated Discovery
EGA	European Genome-phenome Archive
FDR	false discovery rate
FGF	fibroblast growth factor
GEO	NCBI gene expression omnibus
GnRHR	gonadotropin-releasing hormone receptor
HBV	Hepatitis B
HCC	hepatocellular carcinoma
HCV	Hepatitis C
HR	hazard rate
IAP	inhibitor of apoptosis protein
IGF	insulin-like growth factor
IHC	immunohistochemistry
LC	liver cancer
miRNA	micro RNA
OS	overall survival
PANTHER	Protein ANalysis THrough Evolutionary Relationships
PCR	polymerase chain reaction
PDGF	platelet-derived growth factor
PVTT	portal vein tumour thrombosis
RB	retinoblastoma
TCGA	The Cancer Genome Atlas
TKI	receptor tyrosine kinase inhibitor
VEGF	vascular endothelial growth factor

Most HCC patients are diagnosed at an intermediate or advanced stage, rendering only 30% suitable for a potentially curative therapy [7]. Since 2007, patients with an advanced stage HCC (with vascular invasion and extrahepatic spread) can be treated with sorafenib, a multitarget receptor tyrosine kinase inhibitor (TKI). Sorafenib treatment is associated with increased median overall survival (OS) (7.9 versus 10.7 months) [8]. In the Asia Pacific study, 6.5 months OS was reported after sorafenib compared to 4.2 months after placebo [9]. Notably, the modest survival advantage was coupled with frequent side effects and with no increase in quality of life. Lenvatinib, an inhibitor of VEGF, FGF and PDGF receptors and also an RET and KIT inhibitor became recently approved as a first-line treatment for unresectable HCC [10]. Regorafenib (fluoro-sorafenib), another oral TKI, became approved as a second-line treatment for patients progressing on sorafenib. Regorafenib significantly improved both overall (7.8 versus 10.6 months) and progression free survival (1.5 versus 3.1 months) compared to placebo [11]. However, potent adjuvant therapies after surgery are still lacking, and only palliative care is available for patients with multiple metastases, with an estimated OS of less than three months.

HCC is characterized by extreme phenotypic and molecular heterogeneity, and molecular stratification has not yet been established [12]. An HCC can harbour 40–80 mutations, among those five to eight driver mutations per tumour [12]. Cytotoxic chemotherapies fail as ineffective or extremely toxic on already damaged cirrhotic livers. Prognosis prediction is based on clinicopathological parameters including tumour burden, proliferation markers, vascular invasion, liver function and overall health. Ongoing studies now incorporate biomarker candidates in clinical trials to test agents on patients who are most likely to benefit [13]. New single-gene prognostic marker candidates based on low-throughput technologies (e.g. polymerase chain reaction (PCR), immunohistochemistry (IHC)) appear in the literature almost on a daily basis. However, the generalizability of such markers remains questionable.

Vascular invasion of the portal or hepatic veins correlates strongly with HCC recurrence. Following liver transplantation, a 4.4-fold increased risk of HCC recurrence was present in patients with microvascular invasion [14]. At the same time, latent microscopic vascular invasion cannot be detected by pre-operative imaging [15]. Based on autopsy results, 40% of patients with tumours smaller than 5 cm already developed portal vein thrombi [16]. Therefore, identification of molecular changes that correlate with vascular invasion to ascertain the risk of HCC recurrence is a top priority to predict long-term outcome.

In the pursuit of robust prognostic genes, we conducted a meta-analysis by searching the literature for studies focusing on genes associated with OS. To cross-validate these biomarker candidates, we used a large available transcriptomic dataset representing roughly equally two ethnic groups (Asian and White/Caucasian). Prognostic potential for each gene was assessed in a univariate analysis within each ethnic cohort and the strongest markers were included in a multivariate regression. In addition, we also evaluated biomarkers associated with vascular invasion to validate their predictive potential in two independent datasets.

## 2. Material and methods

### 2.1. Identification of previously published biomarker candidates

A PubMed (http://www.pubmed.com/) search conducted in June 2017 using the keywords ‘hepatocellular’, ‘carcinoma’, ‘overall’ and ‘survival’ resulted in 22 999 hits and for terms ‘hepatocellular’, ‘carcinoma’, ‘biomarker’ and ‘mRNA’ in 1615 hits. The overlapping articles of the two sets established the database for cross-validation. All papers were included back to 1998. Only papers written in English were considered in the final biomarker selection. Only research articles were selected, excluding reviews. Biomarkers were retrieved only from studies involving patients undergoing surgical resection or liver transplantation. Only studies reporting molecular differences between tumour and healthy tissue were included in the final list, rejecting papers describing molecular differences between pre- or post-surgery serum or plasma levels.

As survival was frequently associated only with protein expression assessed by IHC or immunoblot, our analysis focusing only on studies reporting mRNA expression had to be extended to include protein-based assays as well. Studies not reporting follow-up data were excluded, same as for papers reporting non-significant associations with survival. Of note, some of the articles assessed the simultaneous co-expression of two to five genes, labelled as composed-biomarkers.

### 2.2. Transcriptomic dataset to validate prognostic biomarkers

The Gene Expression Omnibus (GEO), the European Genome-phenome Archive (EGA) and The Cancer Genome Atlas (TCGA) repositories were screened for datasets with available survival data and at least 30 patients. Accessible transcriptomic data of 371 patients diagnosed with HCC were obtained from the TCGA (https://cancergenome.nih.gov/), with OS available for 364 patients [17]. Most patients were diagnosed at stage I and 67% were male. Patients with Asian and White/Caucasian ethnicity made up the majority, roughly in equal proportions. There was no significant difference in survival when comparing Asian and White/Caucasian patients (*p* = 0.32). Detailed description of the sample population is included in table 2.

**Table 2.** Characterization of patient populations obtained from three independent transcriptomic datasets used in this study to validate biomarkers of overall survival and vascular invasion.

cohort	RNA-seq	Illumina	Affymetrix
source	TCGA	GSE20017	GSE9843
platform	Illumina HiSeq 2000	Illumina HumanRef-8 WG-DASL v. 3.0	Affymetrix HGU133 Plus 2.0 Array
total *n*	371	135	91
gender			
males	250	102	54
females	121	33	27
NA	—	—	10
stage			
stage I	171	—	9
stage II	86	—	56
stage III	85	—	7
stage IV	5	—	8
NA	—	135	11
race			
White/Caucasian	184	102	72
Black or African-American	17	4	3
Asian	158	28	4
vascular invasion	—	40	45
death event	130	32	23

### 2.3. Biomarker candidates associated with vascular invasion

After the initial PubMed search for ‘HCC’ + ‘biomarkers’, the list of genes was further narrowed by keywords ‘vascular invasion’ and ‘PVTT’ (as for portal vein tumour thrombosis). Papers written in non-English and reviews were omitted from the final biomarker selection. The final list consists of studies reporting significant associations between mRNA/protein expression and vascular invasion/PVTT.

### 2.4. Transcriptomic datasets to validate predictive biomarkers

The GEO database was searched for keywords ‘hepatocellular’ + ‘carcinoma’ + ‘vascular’ + ‘invasion’ in May 2017. The search resulted in 18 hits in humans. Of these, 13 remained after filtering for expression profiling by the array. Of these, only two datasets (GSE9843 and GSE20017) had at least 50 patients. GSE9843 contained fresh frozen tissue samples from 91 patients, including 45 with and 34 without vascular invasion [7]. Of all patients, 91% were of White/Caucasian origin, 65.8% were male and 65 patients had published stage (table 2). In GSE20017, genome-wide gene expression profiling from formalin-fixed paraffin-embedded tissues of 135 patients was published. Of all patients, 75.6% were males, 102 of White/Caucasian origin and 40 patients were diagnosed with vascular invasion (table 2). The preprocessed and normalized gene expression data were used in both datasets.

### 2.5. Statistical analyses

A unique HUGO identifier was assigned to each biomarker candidate. Differences in OS were tested by Cox proportional hazards regression. Analyses including Kaplan–Meier survival plots, hazard rates with 95% confidence interval and log-rank *p-*values were calculated and plotted in R using Bioconductor packages separately for the Asian and White/Caucasian ethnic groups and also for the pooled dataset. We considered a *p-*value of 0.05 as significant. The *p-*values from univariate analysis were corrected for multiple testing by computing the false discovery rate (FDR) as described previously [18]. We made a multivariate analysis to compare significant genes to available clinical variables. The expression level of genes was compared with a Mann–Whitney *U*-test between patients with or without vascular invasion independently in both datasets.

## 3. Results

### 3.1. Identification of previously described HCC biomarker candidates

The PubMed search for HCC prognostic biomarkers resulted in 533 hits, of which 513 were written in English, and seven papers were reviews. Evaluation of the remaining 506 papers resulted in 355 relevant articles describing differences between mRNA and/or protein expression and survival. For some genes, multiple references were available, decreasing the number of individual biomarkers to 318, from which overexpression of 194 genes was associated with poor OS. Only 17 papers relied on samples from White/Caucasian subjects describing 21 separate biomarkers. All the other studies involved patients from Asia, mainly from China but also from Korea, Taiwan and Japan.

Molecular association with survival relied frequently on protein expression. In fact, 57% of studies compared survival between groups with low versus high protein expression as measured by IHC, 38% used mRNA expression and the remaining 5% of studies exploited previously available datasets.

Thirteen papers explored the simultaneous expression of two to five genes, thus the cross-validation was conducted on 305 unique genes and 13 composed-biomarkers. The entire gene list with the corresponding manuscript identifier PMIDs is included in electronic supplementary material, table S1.

### 3.2. Prognostic biomarker candidates in Asian patients

**Figure 1.**
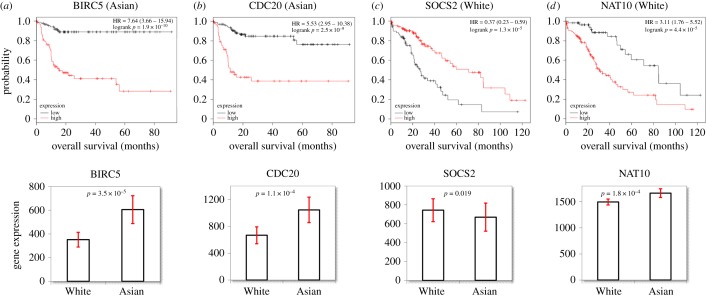
Survival for the two best performing genes in the Asian (*a,b*) and in the White/Caucasian cohorts (*c*,*d*). The expression of all four genes was significantly different between the Asian and White/Caucasian cohorts (bottom subfigures). Red bars: 95% confidence intervals.

Out of the 318 candidate markers, 180 were associated with OS at *p* < 0.05 (electronic supplementary material, table S2). In the pursuit of the strongest biomarkers, a rigorous correction for multiple testing conducted implementing a 0.1% FDR cut-off resulted in 40 significant biomarkers, consisting of 39 unique genes and a single composed-biomarker. High expression of eight genes was associated with improved survival, and expression of 32 biomarkers was linked to poor OS (table 3). Survival plots for the two best performing genes are illustrated in figure 1*a,b*.

**Table 3.** List of 40 significant biomarker candidates associated with overall survival at 0.1% FDR in the Asian cohort. Cox univariate regression analysis. HR, hazard rate; CI, confidence interval.

symbol	gene name	overall survival HR (95% CI), *p*	worse prognosis (expression low–high)	references
*BIRC5*	baculoviral IAP repeat containing 5	HR = 7.64 (3.66–15.94) *p* = 1.9 × 10^−10^	high	[19–21]
*CDC20*	cell division cycle 20	HR = 5.53 (2.95–10.38) *p* = 2.5 × 10^−9^	high	[22]
*PLK1*	Polo-like kinase 1	HR = 6.02 (3.08–11.78) *p* = 3 × 10^−9^	high	[23]
*ALDH2*	aldehyde dehydrogenase-2	HR = 0.19 (0.1–0.35) *p* = 4.2 × 10^−9^	low	[24]
*CCNB1*	cyclin B1	HR = 7.09 (3.29–15.29) *p* = 6.3 × 10^−9^	high	[25]
*FOXK2*	forkhead box K2	HR = 6.83 (3.16–14.73) *p* = 1.5 × 10^−8^	high	[26]
*KIF18A*	kinesin family member 18A	HR = 4.9 (2.66–9.01) *p* = 1.9 × 10^−8^	high	[27]
*BUB1B*	BUB1 mitotic checkpoint serine/threonine kinase B	HR = 4.85 (2.64–8.92) *p* = 2.4 × 10^−8^	high	[28]
*MELK*	maternal embryonic leucine zipper kinase	HR = 5.64 (2.84–11.22) *p* = 3.1 × 10^−8^	high	[29]
*KIAA1524*	cancerous inhibitor of protein phosphatase 2A	HR = 4.71 (2.57–8.63) *p* = 4.0 × 10^−8^	high	[30]
*PTTG1*	pituitary tumour-transforming 1	HR = 7.67 (3.24–18.2) *p* = 5.2 × 10^−8^	high	[31]
*CCNF*	cyclin F	HR = 4.72 (2.54–8.76) *p* = 7.1 × 10^−8^	high	[32]
*PKM2*	pyruvate kinase M2	HR = 4.48 (2.45–8.18) *p* = 1 × 10^−7^	high	[33,34]
*DEPDC1*	DEP domain containing 1	HR = 4.74 (2.47–9.1) *p* = 2.6 × 10^−7^	high	[35]
*STMN1 and SPP1*	stathmin 1 and secreted phosphoprotein 1 (osteopontin)	HR = 4.31 (2.34–7.92) *p* = 3.2 × 10^−7^	high	[36]
*ANGPT2*	angiopoietin 2	HR = 4.25 (2.32–7.76) *p* = 3.3 × 10^−7^	high	[37]
*EZH2*	enhancer of zeste 2 polycomb repressive complex 2 subunit	HR = 5.51 (2.64–11.51) *p* = 3.5 × 10^−7^	high	[38]
*STMN1*	stathmin 1	HR = 4.97 (2.5–9.88) *p* = 4.2 × 10^−7^	high	[39]
*CDC25A*	cell division cycle 25A	HR = 4.22 (2.28–7.8) *p* = 6.7 × 10^−7^	high	[40]
*SLC22A1*	solute carrier family 22 member 1	HR = 0.24 (0.13–0.45) *p* = 8.4 × 10^−7^	low	[41]
*AURKA*	aurora kinase A	HR = 4.5 (2.33–8.66) *p* = 9.6 × 10^−7^	high	[42]
*SEC62*	SEC62 homologue, preprotein translocation factor	HR = 0.25 (0.14–0.46) *p* = 1.1 × 10^−6^	low	[25]
*FOXM1*	forkhead box M1	HR = 4.5 (2.31–8.78) *p* = 1.4 × 10^−6^	high	[43]
*ADH4*	alcohol dehydrogenase 4 (Class II), Pi polypeptide	HR = 0.26 (0.14–0.47) *p* = 1.8 × 10^−6^	low	[44]
*MKI67*	marker of proliferation Ki-67	HR = 3.89 (2.13–7.12) *p* = 2.2 × 10^−6^	high	[22,45]
*MAD2L1*	MAD2 mitotic arrest deficient-like 1 (yeast)	HR = 9.92 (3.06–32.14) *p* = 2.6 × 10^−6^	high	[46]
*CDK4*	cyclin dependent kinase 4	HR = 6.8 (2.68–17.28) *p* = 3.1 × 10^−6^	high	[47]
*CKAP2*	cytoskeleton associated protein 2	HR = 3.64 (2.01–6.61) *p* = 5.5 × 10^−6^	high	[48]
*IQGAP2*	IQ motif containing GTPase activating protein 2	HR = 0.2 (0.09–0.44) *p* = 7.3 × 10^−6^	low	[49]
*SOCS2*	suppressor of cytokine signalling 2	HR = 0.26 (0.13–0.49) *p* = 7.7 × 10^−6^	low	[50]
*E2F1*	E2F transcription factor 1	HR = 8.66 (2.68–28.02) *p* = 1.4 × 10^−5^	high	[39]
*RRM2*	ribonucleotide reductase regulatory subunit M2	HR = 3.7 (1.96–6.98) *p* = 1.6 × 10^−5^	high	[22]
*HMGA1*	high mobility group AT-Hook 1	HR = 3.38 (1.86–6.13) *p* = 2.1 × 10^−5^	high	[51]
*E2F3*	E2F transcription factor 3	HR = 4.1 (2.02–8.31) *p* = 2.3 × 10^−5^	high	[52]
*KIT*	KIT proto-oncogene receptor tyrosine kinase	HR = 3.34 (1.84–6.05) *p* = 2.5 × 10^−5^	high	[53]
*CKS1B*	CDC28 protein kinase regulatory subunit 1B	HR = 4.06 (2–8.23) *p* = 2.6 × 10^−5^	high	[54]
*FAM83D*	family with sequence similarity 83 member D	HR = 3.58 (1.89–6.78) *p* = 2.8 × 10^−5^	high	[55]
*CENPH*	centromere protein H	HR = 4.05 (1.99–8.21) *p* = 2.8 × 10^−5^	high	[56]
*PTEN*	phosphatase and tensin homologue	HR = 0.3 (0.16–0.55) *p* = 3.1 × 10^−5^	low	[57]
*HLX*	H2.0 like homeobox	HR = 0.26 (0.13–0.52) *p* = 3.4 × 10^−5^	low	[58]

**Figure 2.**
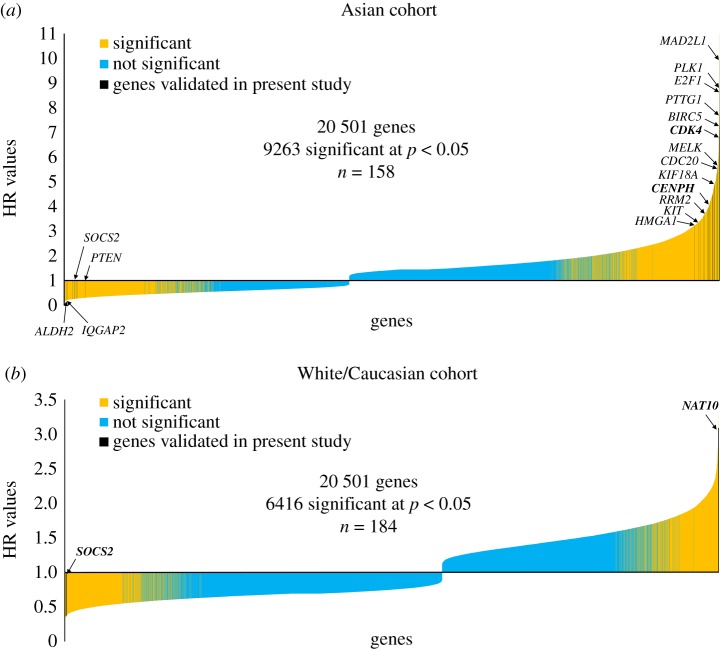
Ranked hazard rates across all 20 501 genes measured by RNA-seq in the Asian (*a*) and in the White/Caucasian cohort (*b*). Genes significantly associated with overall survival are yellow-coloured. The 39 strongest single-gene biomarker candidates validated in the Asian cohort are indicated by black lines including names for selected genes—notably, all positioned among the strongest markers of overall survival. Genes significant after multivariate analysis in the Asian cohort are indicated in bold face, as well as the two biomarker candidates significant at 5% FDR in the White/Caucasian cohort.

To assess the robustness of our meta-analysis, we calculated the hazard rate for each of the 20 501 genes with available expression data to compute their association with OS. Importantly, of the genes identified by the literature search, the ones that remained significant after our validation were located among the strongest markers of survival (figure 2*a,b*).

### 3.3. Gene ontology analysis of prognostic biomarker candidates

The 40 potential prognostic biomarkers of the Asian cohort were subjected to gene enrichment analysis by Database for Annotation, Visualization and Integrated Discovery (DAVID) Bioinformatics Resources 6.8 to gauge the biological meaning of functionally related gene groups [59]. Gene sets related to various facets of cell cycle and cell division, such as sister chromatid cohesion, mitotic nuclear division and cyclin degradation were enriched significantly among markers of the Asian cohort, grouped by relevant function in electronic supplementary material, table S3.

### 3.4. Prognostic biomarker candidates in White/Caucasian patients

Out of 318 biomarkers, 128 were associated with OS at *p* < 0.05 in White/Caucasian subjects (electronic supplementary material, table S2). Out of 21 biomarkers that were appraised originally at White/Caucasian patients only, 10 remained significant at *p* < 0.05: *THOC5* (*p* = 0.00018), *PLAT* (*p* = 0.0012), *HMGA1* (*p* = 0.0025), *PRKDC* (*p* = 0.0096), *SLC22A1* (*p* = 0.013), *CDC20* (*p* = 0.016), the composed-biomarker *CD274-CXCL12* (*p* = 0.019), *CXCL12* (*p* = 0.027), *RRM2* (*p* = 0.045) and *ELAVL1* (*p* = 0.049). However, no biomarker candidates remained significant at 0.1% FDR. After a more lenient correction for multiple testing (at 5% FDR), two genes retained significance. Low expression of *SOCS2* (HR = 0.37, 95% CI = 0.23–0.59, *p* = 1.3 × 10^−5^) while elevated expression of *NAT10* (HR = 3.11, 95% CI = 1.76–5.52, *p* = 4.4 × 10^−5^) was coupled with the poor OS (figure 1*c,d*).

### 3.5. Overlap in significant biomarker candidates between Asian and White/Caucasian cohorts

Out of the 318 biomarker candidates, 226 were significant either in the Asian or in the White/Caucasian cohort at *p* < 0.05. Out of them, 98 and 46 biomarkers were unique to Asian and White/Caucasian patients, respectively, and 82 biomarker candidates were shared by both ethnic groups (electronic supplementary material, table S2). Among the shared biomarkers, 72 were originally described in Asian and 10 in White/Caucasian subjects. Although *NAT10* was not among the 40 significant biomarkers associated with OS in the Asian cohort after setting the threshold at 0.1% FDR, *SOCS2* was among the strongest biomarkers in both ethnic groups.

**Figure 3.**
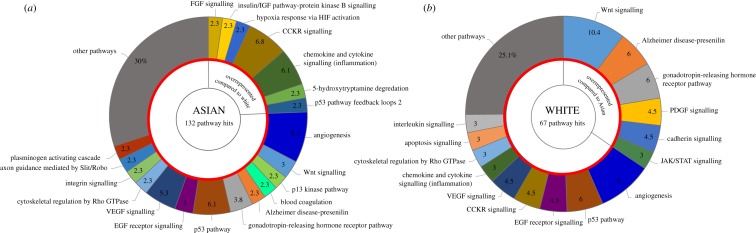
Comparison of gene ontology pathways in Asian (*a*) and White/Caucasian (*b*) patients based on PANTHER gene classification system. All genes significant in the univariate analysis are included. The total number of pathway hits (132 and 67 in the Asian and White cohorts, respectively) represent 100%. Emphasized are those functions that are overrepresented by at least 2% in a given ethnic group compared to the other cohort.

Significant biomarker candidates from univariate analysis of each ethic group were subjected to gene list analysis by the PANTHER (Protein ANalysis THrough Evolutionary Relationships, http://pantherdb.org) gene classification system [60]. Using the pathway classification function, the total number of pathway hits were 132 and 67 in the Asian and White cohorts, respectively. Pathways represented by at least three genes in the Asian and by at least two genes in the White/Caucasian cohort are illustrated in figure 3. Genes participating in FGF-, IGF-signalling, hypoxia–response, CCKR-, chemokine- and cytokine-signalling pathways were overrepresented in the Asian population compared to Caucasians, while genes participating in Wnt-signalling, Alzheimer disease-, presenilin-, PDGF-, cadherin-, JAK/STAT- and GnRHR-signalling pathways were more heavily represented in the Caucasian cohort.

### 3.6. Prognostic biomarker candidates in the pooled dataset

OS did not differ among ethnic groups (*p* = 0.32). After merging patient data with different ethnicity, 178 genes remained associated with OS at *p* < 0.05. Only 40 of these markers reached significance for OS in the analysis at 0.1% FDR, incorporating a single composed-biomarker of two genes and 39 unique genes. High expression of 17 genes correlated with a better outcome, while the high expression of 23 biomarker candidates indicated poor OS (electronic supplementary material, table S4).

### 3.7. Multivariate analysis

In the multivariate analysis, we included parameters which were available for the majority of patients. We had to exclude other clinically relevant features like differentiation, size, cirrhosis and alpha-fetoprotein (AFP) levels because these were only published for a few patients—a robust multivariate analysis can only take account of patients where all data are simultaneously available. In Asian samples, multivariate analysis for OS included stage, sex and expression of 39 unique single-gene biomarkers significant at 0.1% FDR. OS was strongly associated with stage (*p* = 0.0018). Two genes remained prognostic for OS: *CENPH* (*p* = 0.0038) and *CDK4* (*p* = 0.038). Marginal significance was reached by *CDC20* (*p* = 0.053). High expression of all three genes (*CENPH, CDK4* and *CDC20*) predicted poor OS.

In the Caucasian cohort, only two genes remained significant after correction for multiple hypothesis testing (5% FDR); therefore, multivariate analysis included stage, sex and expression of these two genes, *SOCS2* and *NAT10*. OS was associated with the expression of both *SOCS2* (*p* = 0.046) and *NAT10* (*p* = 0.031), but stage and sex were not prognostic.

In the pooled dataset, multivariate analysis for OS including stage, sex and expression of 39 unique, single-gene biomarkers resulted in a significant association between OS and stage (*p* = 2.06 × 10^−5^) and the expression of eight genes: *SPP1* (*p* = 0.0002), *PKM2* (*p* = 0.0008), *EZH2* (*p* = 0.0017), *KIF18A* (*p* = 0.0061), *DEPDC1* (*p* = 0.0092), *CCNF* (*p* = 0.019), *SLC2A1* (*p* = 0.036) and *WASF2* (*p* = 0.048). Increased expression of all eight genes was associated with poor survival outcome.

### 3.8. Biomarkers predictive of vascular invasion

The keywords, vascular ‘invasion’ and ‘PVTT’ narrowed the list of previously filtered 506 papers to 66 and 8 hits, respectively, scaling down our original list of biomarker candidates to 52 unique genes (electronic supplementary material, table S5).

**Figure 4.**
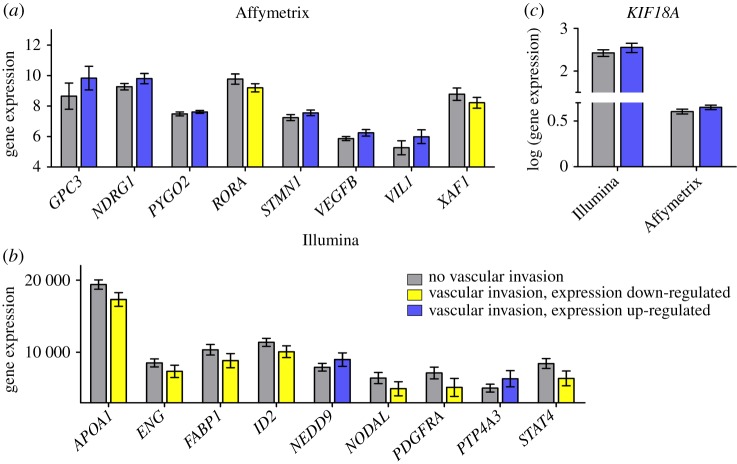
Genes differently expressed between patients with or without vascular invasion at *p* < 0.05 in the Affymetrix (*a*) and Illumina datasets (*b*). In Affymetrix, *RORA* and *XAF1* were downregulated (yellow) with the remaining genes upregulated in the presence of vascular invasion (blue). In Illumina, *NEDD9* and *PTP4A3* (blue) along with *KIF18A* showed increased expression in patients with vascular invasion. *KIF18A* was the singular common gene upregulated in patients with vascular invasion in both datasets (*c*). Error bars: 95% confidence intervals.

In GSE9843, information about the presence of vascular invasion was available for 79 patients, out of which 45 patients presented with vascular invasion. Out of the 52 biomarker candidates, the expression of 9 genes was significantly different between patients with or without vascular invasion at *p* < 0.05 (*GPC3*, *KIF18A*, *NDRG1*, *PYGO2*, *RORA*, *STMN1*, *VEGFB*, *VIL1* and *XAF1*) (table 4) and an additional 4 genes reached marginal significance (*CADM2*, *FAM83D*, *NAA10* and *PEBP1*). The expression of *GPC3*, *KIF18A*, *NDRG1*, *PYGO2, STMN1*, *VEGFB* and *VIL1* was increased and the expression of *RORA* and *XAF1* was decreased in patients with vascular invasion (figure 4*a*).

**Table 4.** Genes associated with the presence of vascular invasion based on the Affymetrix and Illumina datasets. Genes were differently expressed between patients with or without vascular invasion at *p* < 0.05. *KIF18A* is the only gene significant in both datasets.

symbol	gene name	Affymetrix *p*-value	Illumina *p*-value
*KIF18A*	kinesin family member 18A	0.003	0.025
*GPC3*	glypican-3	0.022	n.s.
*NDRG1*	N-Myc downstream regulated 1	0.041	n.s.
*PYGO2*	pygopus family PHD finger 2	0.03	n.s.
*RORA*	RAR-related orphan receptor A	0.005	n.s.
*STMN1*	stathmin 1	0.022	n.s.
*VEGFB*	vascular endothelial growth factor B	0.014	n.s.
*VIL1*	villin-1	0.022	n.s.
*XAF1*	XIAP associated factor 1	0.039	n.s.
*APOA1*	apolipoprotein A1	n.s.	0.0002
*ENG*	endoglin	n.s.	0.028
*FABP1*	fatty acid binding protein 1	n.s.	0.043
*ID2*	inhibitor of DNA binding 2, HLH protein	n.s.	0.01
*NODAL*	nodal growth differentiation factor	n.s.	0.029
*PDGFRA*	platelet-derived growth factor receptor alpha	n.s.	0.004
*STAT4*	signal transducer and activator of transcription 4	n.s.	0.001
*NEDD9*	neural precursor cell expressed, developmentally downregulated 9	n.s.	0.025
*PTP4A3*	protein tyrosine phosphatase type IVA, member 3	n.s.	0.048

In GSE20017, 40 out of 135 patients were diagnosed with vascular invasion. Expression of 10 genes (*APOA1*, *ENG*, *FABP1*, *ID2*, *KIF18A*, *NEDD9*, *NODAL*, *PDGFRA*, *PTP4A3*, *STAT4*) was significantly different between patients with and without vascular invasion at *p* < 0.05 (table 4), and expression difference of *ECM1* and *RPS19BP1* reached marginal significance. The expression of *APOA1, ENG*, *FABP1*, *ID2*, *NODAL*, *PDGFRA* and *STAT4* was decreased and the expression of *KIF18A*, *NEDD9* and *PTP4A3* was increased in patients with vascular invasion (figure 4*b*).

*KIF18A* was the only common gene across the two datasets significantly different correlated to vascular invasion (table 4) and its expression was significantly higher in patients with vascular invasion in both datasets (figure 4*c*).

## 4. Discussion

The lack of targeted therapies in HCC coupled with escalating incidence called forth a paradigm shift. After a decade of phase III failures, current trials started to incorporate molecular markers, and targeted treatments are offered to preselected patients [13]. To accommodate the needs, abundant hypothesis-driven prognostic biomarkers are published in the literature, mostly assessing the transcriptome or proteome by low-throughput technologies (qPCR, IHC, immunoblot). We aimed to cross-validate these published markers to gauge their clinical generalizability. We analysed 318 markers disclosed as related to HCC prognosis in studies published back to 1998. The strongest candidates were also subjected to a multivariate regression. Vascular invasion is one of the strongest clinicopathological features predicting long-term outcome after resection or transplantation. Fifty-two genes associated with vascular invasion were validated in two independent datasets.

Our results highlight the importance of independent cross-validations, as from 318 markers only 180 and 128 genes remained prognostic in the Asian and White/Caucasian cohorts, respectively. Only 40 markers retained significance after rigorous correction for multiple comparisons in the Asian cohort, and none remained significant in the White/Caucasian cohort. This extremely high attrition rate is partially the consequence of small sample sizes in the original studies. Furthermore, in many instances, the transparent and complete reporting allowing the evaluation of the soundness of the study still did not reach the required standards set by the REMARK guidelines [2]. We have to note that survival was frequently assessed as a function of protein expression not inevitably congruent with the transcriptome.

Identifying major components of vascular invasion is vital for a successful therapeutic intervention. Out of the 52 genes described in the literature, 9 and 10 biomarkers were significantly differentially expressed between patients with or without vascular invasion in GSE9843 and GSE20017, respectively. However, of the 18 significant genes, *KIF18A* was the solitary common gene across both datasets. *KIF18A* also appeared among the 40 strongest biomarkers of survival in the Asian cohort. *KIF18A* mediates transport of organelles, proteins and plays a role in microtubule motor activity and mitotic chromosome alignment during cell division [61]. *KIF18A* has also been associated with metastasis in solid tumours (e.g. breast cancer [62]). Specific kinesin motor proteins and molecules participating in cell cycle can be potentially targeted [63].

In the presence of vascular invasion, most biomarkers were overexpressed in GSE9843, but downregulated in GSE20017. One additional strong prognostic biomarker, *STMN1*, was also overexpressed in patients with vascular invasion; however, only in one of the datasets. The lack of overlap highlights the heterogeneity of HCC samples across different datasets, questions the generalizability of the studied biomarkers and emphasizes the importance of external validation.

*SPP1* (osteopontin) was the strongest biomarker candidate associated with survival in the pooled dataset; moreover, *SPP1* expression was significantly associated with worse outcome both in the Asian and White/Caucasian datasets. *SPP1* is a multitasking, highly phosphorylated extracellular matrix protein affecting ECM degradation, cell motility and adhesion, cytoskeletal rearrangement, mitosis and inflammation [64,65]. *SPP1* has been previously recognized as a potential marker of early recurrence and poor prognosis and as a leading metastasis-related gene in HCC [66,67]. A meta-analysis including seven studies confirmed that plasma *SPP1* elevation and AFP-based results have comparable diagnostic performance [68,69], although elevated *SPP1* may be linked to other malignancies, therefore should be combined with other HCC-specific biomarkers [70]. Numerous *SPP1* products are generated by genetic polymorphisms, alternative spicing and posttranslational modifications [65]. Various preclinical therapeutic approaches are being evaluated to target *SPP1*, although when interfering with its normal function severe adverse effects may develop [71].

In the Asian cohort, the strongest biomarkers of survival were particularly enriched by genes controlling multiple aspects of cell division, including sister chromatid cohesion and positioning (*CENPH, KIF18A*), mitotic checkpoint (*BUB1B, MAD2L1*), cell cycle transitions (*CCNB1, PLK1, FOXM1, BIRC5, AURKA, CDC25A, MELK, CCNF*), G1-phase progression (*CDK4, E2F1*) and cyclin degradation (*CDC20, PTTG1*). Various efforts classified HCC into non-proliferative and proliferative genotypes, with worse outcome in the latter [13]. Our results are congruent with previous findings, where the G3 tumour type overexpressing genes controlling cell cycle was associated with the most severe prognosis [72], and G3 type was confirmed as an independent predictor of recurrence [73].

*BIRC5* (survivin) was the strongest predictor of poor survival based on the univariate analysis in the Asian cohort (figure 1). *BIRC5* is the smallest member of the inhibitor of apoptosis protein (IAP) family, a multitasking protein that regulates mitosis, suppresses apoptosis and affects proliferation, angiogenesis and cellular stress response [74–76]. *BIRC5* is almost entirely absent in adult tissue, but its upregulation is linked to worse survival in numerous solid tumours [77–79]. A systematic meta-analysis confirmed the prognostic role of *BIRC5* gene in HCC across 14 different studies involving 890 patients [80]. The multiple and redundant regulation of *BIRC5* poses a difficulty for inhibitor development and requires simultaneous targeting of multiple networks [81,82]. *BIRC5* deficiency interferes with mitosis, increases TNF*α* and leads to senescence. Promising tumour regression was reported after synergistic effects of a mitotic inhibitor acting on *PLK1* (BI2536) to induce mitotic arrest, and a proapoptotic agent (LCL161) to sensitize HCC cells to TNFα-triggered cell death in a murine model of HCC [83].

Two genes remained independent prognostic factors after multivariate analysis in the Asian cohort: *CENPH* and *CDK4*, both linked to poor prognosis. Centromeres and their associated kinetochores are required for proper spindle attachment, separation of sister chromatids and regulation of mitotic checkpoint activity [84]. The product of *CENPH* is a fundamental component of the inner kinetochore–nucleosome associated complex [85]. Misregulation of centromere and kinetochore protein levels is linked to missegregations, aneuploidy and genome rearrangement [86,87], increasing chromosomal instability (CIN) and tumourigenesis. Accumulating evidence suggests that *CENPH* upregulation has been linked to aneuploidy, high proliferative ability and poor prognosis in various solid tumours [88–90].

*CDK4* acts on the retinoblastoma (RB) signalling pathway, promoting progression from G1 to S phase during the cell cycle. A selective CDK4/6 inhibitor, palbociclib, became recently approved as first-line treatment for oestrogen-positive, HER2-negative breast cancer patients. The majority of HCCs harbour an intact *RB1* gene [91], rendering them potentially sensitive to palbociclib. In HCC, palbociclib suppressed *in vitro* cell proliferation, restricted *in vivo* tumour growth and increased survival with enhanced effects when combined with sorafenib [92].

In the White cohort only genes remained significant after *p-*value correction, possibly as a consequence of most articles focusing at Asian patients; in fact, only 6% of papers analysed samples from White/Caucasian subjects. Of *SOCS2* and *NAT10,* only the latter offers a potential target for future therapies. *NAT10* is involved in histone-acetylation and regulates telomerase activity through TERT-promoter [93]. It is localized mainly in the cell membrane, particularly at the invasive leading edge of tumours, increasing motility and invasive potential that correlates with nuclear β-catenin accumulation [94,95]. *NAT10* also activates p53 in response to DNA damage by acetylation [96]. *NAT10* is the target of a small molecule compound called ‘remodelin’ to treat laminopathies and premature ageing syndromes [97], not yet tested in the context of HCC.

Among our strongest biomarkers of survival, several genes have been repeatedly identified in the process of oncogenic transformation, including the development of LC [98]. Our gene ontology analyses revealed ethnic group-specific signalling pathway activations (figure 3). The differences are probably related to the distinct aetiology of the disease, as viral infections underlie most HCCs in Asia, while in western countries, chronic liver disease related to alcohol consumption and non-alcoholic steatohepatitis is responsible for HCC [6].

Our narrowed list of biomarkers may provide optimal targets to tackle pivotal signalling pathways of HCC including the Wnt/β-catenin, TP53, RAS, JAK–STAT, MAPK, the retinoblastoma protein pRb1, oxidative stress and chromatin remodelling pathways [13]. *PKM2* is the rate-limiting catalytic activator of glycolytic metabolic pathways and also exerts a non-glycolytic function as a transcriptional activator, possibly affecting numerous signalling pathways, including Wnt/β-catenin, JAK-STAT and AKT signalling [99]. P53 inactivation is abundant in HCC even in the absence of *TP53* mutations, leading to overexpression of genes normally repressed by p53 such as *EZH2,* a histone-methyltransferase that epigenetically regulates stem cell maintenance, and *CCNB1*, *E2F1* and *FOXM1,* all present in our list of strongest prognostic markers [17]. Dosage-dependent copy number gains of *E2F1* inactivate the retinoblastoma tumour suppressor pathway, driving HCC development in mice [100].

Several of our strongest prognostic biomarker candidates have also been identified with a functionally significant role in drug response. For example, the tyrosine kinase receptor KIT is a target of the kinase inhibitor sorafenib [101]. Moreover, sorafenib decreases protein levels of survivin, the product of the anti-apoptotic *BIRC5* [102] and expression of numerous genes implicated in cell cycle, DNA replication and cell proliferation (e.g. *CDC20*, *CDC25A*) [103]. Our list of significant prognostic biomarkers might help to select candidates for future drug development.

Altogether, here we aimed to deliver authentication for gene- and protein expression-based HCC biomarkers by assessing the generalizability of previous findings. Integration of copy number variations, somatic mutations, methylation patterns, proteomics and mRNA expression might provide more throughout the identification of factors related to HCC [17]. Additional RNAs complementing mRNA started to emerge as potential biomarkers and therapeutic targets. A recent paper identified 223 miRNAs associated with HCC and validated their significance in OS [104].

## 5. Conclusion

The low reproducibility of the published prognostic biomarkers uncovered in our analysis draws attention to the need for more rigorous research practices in this heterogeneous neoplastic disease with a highly variable aetiology. In summary, we provide a unified pipeline using already available transcriptomic datasets to enable the validation and ranking of future biomarker candidates.

## Supplementary Material

Supplementary Table 1

## Supplementary Material

Supplementary Table 2

## Supplementary Material

Supplementary Table 3

## Supplementary Material

Supplementary Table 4

## Supplementary Material

Supplementary Table 5

## Data accessibility

The datasets used and/or analysed during the current study are available at TCGA (https://cancergenome.nih.gov/) and NCBI Gene Expression Omnibus (GEO) databases (dataset GSE9843 is available at https://www.ncbi.nlm.nih.gov/geo/query/acc.cgi?acc=GSE9843 and dataset GSE20017 is available at https://www.ncbi.nlm.nih.gov/geo/query/acc.cgi?acc=GSE20017).
