# Supplementary material for: Determining consistent prognostic biomarkers of overall survival and vascular invasion in hepatocellular carcinoma
Source: R Soc Open Sci. 2018 Dec 5;5(12):181006. doi: 10.1098/rsos.181006 (PMC6304123; doi:10.1098/rsos.181006)
Supplement: Supplementary Table 1 [file rsos181006supp1.docx]

**Determining consistent prognostic biomarkers of overall survival and vascular invasion in hepatocellular carcinoma**

Otília Menyhárt, Ádám Nagy, Balázs Győrffy

**Supplementary Table 1.**

**The list of 318 biomarkers containing 305 individual genes and 13 composed-biomarkers out of the 355 relevant publications.** Expression levels conferring worse prognosis are based on the original articles, not inevitably congruent with the results of the current analysis.

| **Symbol** | **Gene name** | **Worse prognosis (expression level)** | **PMID** |
| --- | --- | --- | --- |
| ***ABCB1*** | ATP Binding Cassette Subfamily B Member 1 | high | 26261589, 11579380 |
| ***ABCG2*** | ATP Binding Cassette Subfamily G Member 2 (Junior Blood Group) | high | 20442200 |
| ***ACE2*** | angiotensin converting enzyme 2 | low | 25701390 |
| ***ACTA2*** | Actin, Alpha 2, Smooth Muscle, Aorta | low | 25449435 |
| ***ACVR1*** | Activin A Receptor Type 1 | high | 25271001 |
| ***ADAM10*** | ADAM Metallopeptidase Domain 10 | high | 24670536 |
| ***ADH4*** | Alcohol Dehydrogenase 4 (Class II), Pi Polypeptide | low | 22147505 |
| ***ADRB2*** | Adrenoceptor Beta 2 | high | 22588469 |
| ***AFP*** | Alpha Fetoprotein | high | 15754002, 18466280 |
| ***AFP, SPP1*** |  | joint expression | 15754002 |
| ***AGER*** | Advanced Glycosylation End-Product Specific Receptor | high | 21717246 |
| ***AIM2*** | Absent In Melanoma 2 | low | 28580773 |
| ***AJAP1*** | Adherens Junctions Associated Protein 1 | low | 26122373 |
| ***AKAP12*** | A-Kinase Anchoring Protein 12 | low | 22052684 |
| ***AKT1*** | phosphorylated protein kinase B | high | 26932478 |
| ***ALDH1A1*** | Aldehyde dehydrogenase 1A1 | low | 26160842 |
| ***ALDH1L1*** | Aldehyde Dehydrogenase 1 Family Member L1 | low | 21987076 |
| ***ALDH2*** | Aldehyde dehydrogenase-2 | low | 28027570 |
| ***ANGPT2*** | Angiopoietin 2 | high | 17931370 |
| ***ANP32A*** | Acidic Nuclear Phosphoprotein 32 Family Member A | high | 20683644 |
| ***ANPEP*** | Alanyl Aminopeptidase, Membrane | high | 26735577 |
| ***ANXA4*** | Annexin A4 | high | 26779633 |
| ***APOD*** | Apolipoprotein D | low | 15756681 |
| ***ARID1A*** | AT-Rich Interaction Domain 1A | high | 26589513 |
| ***ARL6IP5*** | ADP Ribosylation Factor Like GTPase 6 Interacting Protein 5 | low | 23169062 |
| ***ATF5*** | Activating Transcription Factor 5 | low | 25294425 |
| ***ATOH8*** | atonal homolog 8 | low | 26099525 |
| ***ATXN7*** | ataxin 7 | low | 27855399 |
| ***AURKA*** | Aurora Kinase A | high | 15041727 |
| ***BARX2*** | BARX Homeobox 2 | low | 24716715 |
| ***BATF2*** | Basic Leucine Zipper ATF-Like Transcription Factor 2 | low | 20473897 |
| ***BCL2L11*** | BCL2-Like 11 (Apoptosis Facilitator) | low | 25788265 |
| ***BCL3*** | B-Cell CLL/Lymphoma 3 | high | 26882953 |
| ***BIRC3*** | Baculoviral IAP Repeat Containing 3 | high | 22682366 |
| ***BIRC5*** | Baculoviral IAP Repeat Containing 5 | low | 17559540, 15547736, 12374680 |
| ***BLZF1*** | Basic Leucine Zipper Nuclear Factor 1 | low | 26342799 |
| ***BMP7*** | Bone Morphogenetic Protein 7 | high | 23179403 |
| ***BTG1*** | B‑cell translocation gene 1 | low | 25405901 |
| ***BTG3*** | BTG Anti-Proliferation Factor 3 | low | 24147003 |
| ***BUB1B*** | BUB1 Mitotic Checkpoint Serine/Threonine Kinase B | high | 19861558 |
| ***CADM1*** | Cell Adhesion Molecule 1 | low | 21271221 |
| ***CADM2*** | Cell Adhesion Molecule 2 | low | 24240726 |
| ***CAP2*** | Adenylyl Cyclase-Associated Protein 2 | high | 26500030 |
| ***CBS*** | Cystathionine-Beta-Synthase | low | 19424622 |
| ***CBX4*** | Chromobox 4 | high | 23943028 |
| ***CCNB1*** | Cyclin B1 | high | 22682366 |
| ***CCNF*** | Cyclin F | low | 23305207 |
| ***CCR6*** | C-C Motif Chemokine Receptor 6 | high | 16706828 |
| ***CCRL1*** | Atypical Chemokine Receptor 4 | low | 25255875 |
| ***CD151*** | CD151 Molecule (Raph Blood Group) | high | 19065669 |
| ***CD151, MET*** |  | joint expression | 19065669 |
| ***CD24*** | CD24 Antigen (Small Cell Lung Carcinoma Cluster 4 Antigen) | high | 25271001 |
| ***CD274*** | CD274 Molecule | high | 27913861, 19826049 |
| ***CD274, CXCL12neg*** |  | joint expression | 27913861 |
| ***CD276*** | CD276 Molecule | high | 25370943 |
| ***CD44*** | CD44 Molecule (Indian Blood Group) | high | 25271001, 24647926, 22552294 |
| ***THY1, CD24, PROM1, ANPEP*** |  | joint expression | 26735577 |
| ***CDC20*** | Cell Division Cycle 20 | high | 28246274 |
| ***CDC25A*** | Cell Division Cycle 25A | high | 12738732 |
| ***CDC5L*** | Cell division cycle 5-like | high | 26553251 |
| ***CDCP1*** | CUB domain-containing protein 1 | high | 26307391 |
| ***CDH1*** | Cadherin 1 | high | 24833103 |
| ***CDH1, VIM*** |  | joint expression | 24833103 |
| ***CDK4*** | Cyclin Dependent Kinase 4 | high | 23292829 |
| ***CDK5R2*** | Cyclin Dependent Kinase 5 Regulatory Subunit 2 | low | 20936377 |
| ***CDKN1B*** | Cyclin Dependent Kinase Inhibitor 1B | low | 19866239 |
| ***CDKN1C*** | Cyclin Dependent Kinase Inhibitor 1C | low | 23842948 |
| ***CDKN1C, RHOA*** |  | joint expression | 23842948 |
| ***CDX1*** | Caudal Type Homeobox 1 | low | 27566019 |
| ***CENPH*** | Centromere Protein H | high | 23970101 |
| ***CHI3L1*** | Chitinase 3 Like 1 | high | 23525579 |
| ***CISD2*** | CDGSH iron sulfur domain 2 | high | 26722601 |
| ***CKAP2*** | Cytoskeleton Associated Protein 2 | high | 24238125 |
| ***CKS1B*** | CDC28 Protein Kinase Regulatory Subunit 1B | high | 19866239 |
| ***CKS1B, CDKN1B*** |  | joint expression | 19866239 |
| ***CLDN7*** | Claudin 7 | low | 23146509 |
| ***COL1A1*** | Collagen Type I Alpha 1 Chain | low | 24552139 |
| ***CPE*** | carboxypeptidase E | high | 26803519 |
| ***CTHRC1*** | Collagen Triple Helix Repeat Containing 1 | high | 23922981 |
| ***CTTN*** | Cortactin | high | 24409670, 23518204 |
| ***CXCL12*** | C-X-C Motif Chemokine Ligand 12 | low | 27913861 |
| ***CXCR4*** | C-X-C Motif Chemokine Receptor 4 | high | 25363530 |
| ***CYTH2*** | Cytohesin 2 | high | 23545718 |
| ***DEK*** | DEK Proto-Oncogene | high | 25351213 |
| ***DEPDC1*** | DEP Domain Containing 1 | high | 25605201 |
| ***DHX33*** | DEAH-box helicase 33 | high | 27073163 |
| ***DIXDC1*** | Dishevelled-Axin domain containing 1 | low | 27468723 |
| ***DKC1*** | Dyskerin Pseudouridine Synthase 1 | high | 22912812 |
| ***DLX4*** | Distal-Less Homeobox 4 | high | 24824934 |
| ***DPYSL3*** | Dihydropyrimidinase Like 3 | low | 25173447 |
| ***DUOX1*** | Dual oxidase 1 | low | 27108801 |
| ***E2F1*** | E2F Transcription Factor 1 | high | 22911364 |
| ***E2F3*** | E2F Transcription Factor 3 | high | 24402082 |
| ***ECM1*** | Extracellular Matrix Protein 1 | high | 21128013 |
| ***EDIL3*** | EGF Like Repeats And Discoidin Domains 3 | high | 25273699 |
| ***EFEMP1*** | EGF Containing Fibulin Like Extracellular Matrix Protein 1 | low | 23936443 |
| ***EIF3H*** | eukaryotic translation initiation factor 3 | high | 27340783 |
| ***EIF4EBP1*** | Eukaryotic Translation Initiation Factor 4E Binding Protein 1 | high | 25658620 |
| ***ELAVL1*** | ELAV Like RNA Binding Protein 1 | high | 25678597 |
| ***ENAH*** | Enabled Homolog (Drosophila) | high | 24683008 |
| ***EP300*** | E1A Binding Protein P300 | high | 21205329 |
| ***EPAS1*** | Endothelial PAS Domain Protein 1 | high | 24374892 |
| ***EPCAM*** | epithelial cell adhesion molecule | high | 26932478, 25271001 |
| ***EPHA2*** | EPH Receptor A2 | high | 19642143 |
| ***EPOR*** | Erythropoietin Receptor | low | 23496059 |
| ***ERBB2*** | Erb-B2 Receptor Tyrosine Kinase 2 | high | 19610068 |
| ***EYA4*** | EYA Transcriptional Coactivator And Phosphatase 4 | low | 24306662 |
| ***EZH2*** | Enhancer Of Zeste 2 Polycomb Repressive Complex 2 Subunit | high | 24966962 |
| ***FABP1*** | Fatty Acid Binding Protein 1 | low | 25436304 |
| ***FAM83D*** | Family With Sequence Similarity 83 Member D | high | 26125229 |
| ***FBLN5*** | Fibulin-5 | low | 25494879 |
| ***FERMT1*** | Fermitin Family Member 1 | high | 25592379 |
| ***FERMT2*** | Fermitin Family Member 2 | high | 25618552 |
| ***FLT4*** | Fms Related Tyrosine Kinase 4 | high | 12017318 |
| ***FOXD3*** | forkhead box D3 | low | 26112097 |
| ***FOXF2*** | Forkhead Box F2 | low | 25824262 |
| ***FOXK2*** | Forkhead Box K2 | high | 28506857 |
| ***FOXM1*** | Forkhead Box M1 | high | 21482449 |
| ***FOXP1*** | Forkhead Box P1 | high | 22422806 |
| ***GABARAPL1*** | GABA Type A Receptor Associated Protein Like 1 | low | 24647565 |
| ***GABPA*** | GA Binding Protein Transcription Factor Alpha Subunit | low | 28549418 |
| ***GADD45G*** | Growth arrest DNA damage-inducible gene 45 | low | 26172295 |
| ***GJA1*** | Gap Junction Protein Alpha 1 | low | 23800357 |
| ***GLI1*** | GLI Family Zinc Finger 1 | high | 22911366 |
| ***GLTSCR2*** | Glioma Tumor Suppressor Candidate Region Gene 2 | high | 23532381 |
| ***GMNN*** | Geminin, DNA Replication Inhibitor | high | 28246274 |
| ***GOLM1*** | Golgi Membrane Protein 1 | low | 23607749 |
| ***GOLPH3*** | Golgi Phosphoprotein 3 | high | 25385148, 24867097 |
| ***GPC3*** | Glypican 3 | high | 20964802, 22654434 |
| ***GPRC5A*** | G Protein-Coupled Receptor Class C Group 5 Member A | high | 23632812 |
| ***HACE1*** | HECT domain and ankyrin repeat containing E3 ubiquitin protein ligase 1 | low | 27805249 |
| ***HDGF*** | Hepatoma-Derived Growth Factor | high | 16411141, 14508832 |
| ***HIF1A*** | Hypoxia Inducible Factor 1 Alpha Subunit | high | 24374892, 19948069 |
| ***HIF1A, EPAS1*** |  | joint expression | 24374892 |
| ***HINT2*** | Histidine Triad Nucleotide Binding Protein 2 | low | 16762638 |
| ***HLX*** | H2.0-like homeobox 1 | low | 26631039 |
| ***HMGA1*** | High Mobility Group AT-Hook 1 | high | 27855356 |
| ***HMGB1*** | High Mobility Group Box 1 | high | 22747650 |
| ***HOTAIR*** | HOX Transcript Antisense RNA | high | 21327457 |
| ***HOXB7*** | Homeobox B7 | high | 27272787 |
| ***HPSE*** | Heparanase | low | 22952874 |
| ***HSPA2*** | Heat Shock Protein Family A (Hsp70) Member 2 | high | 25117073 |
| ***ICAM1*** | Intercellular Adhesion Molecule 1 | high | 26667486 |
| ***ID1*** | Inhibitor Of DNA Binding 1, HLH Protein | high | 20574154 |
| ***ID2*** | Inhibitor Of DNA Binding 2, HLH Protein | low | 18281534 |
| ***IGF2BP3*** | Insulin Like Growth Factor 2 MRNA Binding Protein 3 | high | 22211286 |
| ***IGFBP1*** | Insulin Like Growth Factor Binding Protein 1 | low | 25337205 |
| ***IKBKG*** | Inhibitor Of Kappa Light Polypeptide Gene Enhancer In B-Cells, Kinase Gamma | low | 22176836 |
| ***IKZF1*** | IKAROS Family Zinc Finger 1 | low | 25301737 |
| ***IL18R1*** | Interleukin 18 Receptor 1 | high | 16108033 |
| ***IL2*** | Interleukin 2 | low | 20940284 |
| ***IL8*** | C-X-C Motif Chemokine Ligand 8 | high | 25271001 |
| ***IMP3*** | IMP3, U3 Small Nucleolar Ribonucleoprotein | high | 24647926 |
| ***ING3*** | Inhibitor Of Growth Family Member 3 | low | 22550337 |
| ***ING4*** | Inhibitor Of Growth Family Member 4 | low | 19208663 |
| ***INPPL1*** | Inositol Polyphosphate Phosphatase Like 1 | high | 24228114 |
| ***IQGAP1*** | IQ Motif Containing GTPase Activating Protein 1 | high | 24998570 |
| ***IQGAP2*** | IQ Motif Containing GTPase Activating Protein 2 | low | 24998570 |
| ***KAL1*** | Kallmann syndrome-1, Anosmin 1 | low | 25892360 |
| ***KDR*** | Kinase Insert Domain Receptor | high | 21270061 |
| ***KIAA0114*** | Differentiation Antagonizing Non-Protein Coding RNA | high | 25964079 |
| ***KIAA1524*** | Cancerous Inhibitor Of Protein Phosphatase 2A | high | 22847158 |
| ***KIF18A*** | Kinesin Family Member 18A | high | 25431949 |
| ***KIF1B*** | kinesin family member 1B | low | 26217094 |
| ***KISS1, KISS1R*** | KiSS-1 Metastasis-Suppressor and KISS1 Receptor | joint expression | 12898236 |
| ***KIT*** | KIT Proto-Oncogene Receptor Tyrosine Kinase | low | 15617841 |
| ***KRT19*** | Cytokeratin 19 | high | 26588210, 22045674, 20442200 |
| ***L1CAM*** | L1 Cell Adhesion Molecule | high | 22888955 |
| ***LARP1*** | La Ribonucleoprotein Domain Family Member 1 | high | 24159927 |
| ***LIN28A*** | Lin-28 Homolog A | high | 22429493 |
| ***LOXL4*** | Lysyl Oxidase Like 4 | low | 26097573 |
| ***LYVE1*** | Lymphatic Vessel Endothelial Hyaluronan Receptor 1 | low | 24649290 |
| ***MACC1*** | MACC1, MET Transcriptional Regulator | high | 23717574, 21955323 |
| ***MAD2L1*** | MAD2 Mitotic Arrest Deficient-Like 1 (Yeast) | high | 18715617 |
| ***MAGED1*** | Neurotrophin Receptor-Interacting MAGE Homolog | high | 26998088 |
| ***MAGED4*** | MAGE Family Member D4 | high | 24068544 |
| ***MAGI1*** | Membrane Associated Guanylate Kinase, WW And PDZ Domain Containing 1 | low | 21942217 |
| ***MAT1A*** | Methionine Adenosyltransferase 1A | low | 23072598 |
| ***MCAM*** | Melanoma Cell Adhesion Molecule | high | 25728681 |
| ***MELK*** | Maternal Embryonic Leucine Zipper Kinase | high | 27798878 |
| ***MEP1A*** | Meprin A Subunit Alpha | high | 26660154 |
| ***MET*** | MET Proto-Oncogene, Receptor Tyrosine Kinase | high | 19065669 |
| ***MFN2*** | mitofusin-2 | low | 27389277 |
| ***MKI67*** | Marker Of Proliferation Ki-67 | high | 28246274, 21334407 |
| ***MMP12*** | Matrix Metallopeptidase 12 | high | 21683576 |
| ***MMP7*** | Matrix Metallopeptidase 7 | high | 19948069 |
| ***MTOR*** | phosphorylated mammalian target of rapamycin | high | 26932478 |
| ***NAT10*** | N-acetyltransferase 10 | high | 26823802 |
| ***NCL*** | Nucleolin | high | 25230759 |
| ***NDRG1*** | N-Myc Downstream Regulated 1 | high | 17170744 |
| ***NEDD9*** | Neural Precursor Cell Expressed, Developmentally Down-Regulated 9 | high | 25812772 |
| ***NES*** | nestin | high | 20442200 |
| ***NEU1*** | Neuraminidase 1 | high | 27602751 |
| ***NKD1*** | Naked Cuticle Homolog 1 | low | 27507614 |
| ***NKX2-8*** | NK2 Homeobox 8 | low | 24678995 |
| ***NNMT*** | Nicotinamide N-Methyltransferase | high | 19216803 |
| ***NODAL*** | Nodal Growth Differentiation Factor | high | 24465741 |
| ***NOTCH3*** | NOTCH3 | high | 28568708 |
| ***NPAS2*** | Neuronal PAS Domain Protein 2 | high | 28333141 |
| ***NPRL2*** | NPR2-Like, GATOR1 Complex Subunit | low | 19274676 |
| ***NRP1*** | Neuropilin1 | high | 26563279 |
| ***NUAK1*** | NUAK Family Kinase 1 | high | 23516026 |
| ***PARK7*** | Parkinsonism Associated Deglycase | high | 21410067 |
| ***PDCD4*** | Programmed Cell Death 4 | low | 26871813 |
| ***PDCD5*** | Programmed Cell Death 5 | low | 23807738 |
| ***PDGFRA*** | Platelet Derived Growth Factor Receptor Alpha | low | 25333264, 19948069 |
| ***PDK1*** | 3-phosphoinositide-dependent protein kinase-1 | high | 27672330 |
| ***PDSS2*** | Decaprenyl Diphosphate Synthase Subunit 2 | low | 25189544 |
| ***PEBP1*** | Phosphatidylethanolamine Binding Protein 1 | low | 20739083 |
| ***PECAM1*** | Platelet And Endothelial Cell Adhesion Molecule 1 | high | 12017318 |
| ***PEMT*** | Phosphatidylethanolamine N-Methyltransferase | low | 12931022 |
| ***PI4KA*** | Phosphatidylinositol 4-Kinase Alpha | high | 24393405 |
| ***PIWIL1*** | Piwi Like RNA-Mediated Gene Silencing 1 | high | 21989785 |
| ***PKM2*** | Pyruvate kinase M2 | high | 25788265, 24466275 |
| ***PLAT*** | Plasminogen Activator, Tissue Type | high | 9605771 |
| ***PLAU*** | Plasminogen Activator, Urokinase | high | 9605771, 11079728 |
| ***PLAU, PLAUR, SERPINE1*** |  | joint expression | 11079728 |
| ***PLAUR*** | Plasminogen Activator, Urokinase Receptor | high | 9605771, 11079728 |
| ***PLCE1*** | Phospholipase C Epsilon 1 | low | 28031722 |
| ***PLK1*** | Polo Like Kinase 1 | high | 19725153 |
| ***PNKD*** | Myofibrillogenesis regulator 1 or Paroxysmal Nonkinesigenic Dyskinesia | high | 26823810 |
| ***PNLIPRP3*** | Pancreatic Lipase Related Protein 3 | high | 19640199 |
| ***POU5F1*** | POU Class 5 Homeobox 1 | high | 22824146 |
| ***PREX2*** | Phosphatidylinositol-3,4,5-Trisphosphate Dependent Rac Exchange Factor 2 | high | 28205209 |
| ***PRKCA*** | Protein Kinase C Alpha | high | 17459358 |
| ***PRKDC*** | Protein Kinase, DNA-Activated, Catalytic Polypeptide | high | 24136149 |
| ***PRMT5*** | protein arginine methyltransferase 5 | high | 28101581 |
| ***PROM1*** | Prominin-Like Protein 1 | high | 26932478, 21331808, 20442200 |
| ***PRRX1*** | Paired Related Homeobox 1 | low | 25404478 |
| ***PTCH1*** | Patched 1 | high | 22911366 |
| ***PTEN*** | phosphatase and tensin homolog deleted on chromosome 10 | low | 26932478 |
| ***PTGS2*** | Prostaglandin-Endoperoxide Synthase 2 | high | 19948069 |
| ***PTK2*** | Protein Tyrosine Kinase 2 | high | 15246215 |
| ***PTOV1*** | prostate tumor overexpressed 1 | high | 25634174 |
| ***PTP4A3*** | Protein Tyrosine Phosphatase Type IVA, Member 3 | high | 23064776 |
| ***PTTG1*** | Pituitary Tumor-Transforming 1 | high | 16628605 |
| ***PVRL4*** | Nectin Cell Adhesion Molecule 4 | high | 26793002 |
| ***PYGO2*** | Pygopus Family PHD Finger 2 | high | 25545771 |
| ***RASSF10*** | Ras-association domain family 10 | low | 26701853 |
| ***RASSF5*** | Ras Association Domain Family Member 5 | low | 24563371 |
| ***RCHY1*** | Ring Finger And CHY Zinc Finger Domain Containing 1 | high | 19551892 |
| ***REG3A*** | Regenerating Family Member 3 Alpha | low | 15814635 |
| ***REG3A, REG1A*** |  | joint expression | 15814635 |
| ***RELN*** | reelin | low | 20734148 |
| ***RHOA*** | Ras Homolog Family Member A | high | 23842948, 16806792 |
| ***RHOC*** | Ras Homolog Family Member C | high | 15150600 |
| ***RIPK1*** | receptor-interacting protein kinase 1 | high | 27699664 |
| ***ROR2*** | Receptor Tyrosine Kinase Like Orphan Receptor 2 | low | 22493546 |
| ***RORA*** | RAR Related Orphan Receptor A | low | 24798975 |
| ***RPS19BP1*** | Ribosomal Protein S19 Binding Protein 1 | high | 26339164 |
| ***RRAD*** | RRAD, Ras Related Glycolysis Inhibitor And Calcium Channel Regulator | low | 26546438 |
| ***RRM2*** | Ribonucleotide Reductase Regulatory Subunit M2 | high | 28246274 |
| ***RUNX2*** | Runt-related transcription factor 2 | high | 27666365 |
| ***S100A14*** | S100 Calcium Binding Protein A14 | high | 23886191 |
| ***SAMSN1*** | SAM domain, SH3 domain, and nuclear localization signals 1 | low | 25805236 |
| ***SEC62*** | SEC62 Homolog, Preprotein Translocation Factor | high | 22682366 |
| ***SERPINE1*** | Serpin Family E Member 1 | high | 11079728 |
| ***SFRP1*** | Secreted frizzled-related protein-1 | low | 26851021 |
| ***SIRT1*** | Sirtuin 1 | high | 22146883 |
| ***SKP2*** | S-Phase Kinase Associated Protein 2 | high | 19866239 |
| ***SLC22A1*** | Solute Carrier Family 22 Member 1 | low | 22439694 |
| ***SLC2A1*** | Solute Carrier Family 2 Member 1 | high | 21334407 |
| ***SLC39A6*** | Solute Carrier Family 39 Member 6 | high | 26684241 |
| ***SLC5A8*** | Solute Carrier Family 5 Member 8 | low | 27465549 |
| ***SLC7A11*** | Solute Carrier Family 7 Member 11 | high | 23229496 |
| ***SLC7A5*** | Solute Carrier Family 7 Member 5 | high | 23696029 |
| ***SLC9A1*** | Solute Carrier Family 9 Member A1 | high | 19876777 |
| ***SMARCA2*** | SWI/SNF Related, Matrix Associated, Actin Dependent Regulator Of Chromatin, Subfamily A, Member 2 | low | 23088494 |
| ***SMARCB1*** | SWI/SNF Related, Matrix Associated, Actin Dependent Regulator Of Chromatin | low | 27111394 |
| ***SOCS2*** | Suppressor Of Cytokine Signaling 2 | low | 27465557 |
| ***SOCS3*** | Suppressor Of Cytokine Signaling 3 | low | 22977555 |
| ***SOX1*** | Sex-determining region Y (SRY)-box 1 | low | 26191244 |
| ***SPC24*** | SPC24, NDC80 Kinetochore Complex Component | high | 26515591 |
| ***SPHK1*** | Sphingosine Kinase 1 | high | 28454397 |
| ***SPIB*** | Spi-B Transcription Factor | high | 26610895 |
| ***SPP1*** | Secreted Phosphoprotein 1 (Osteopontin) | low | 25449435, 17161983, 16739096, 15754002 |
| ***SPP1, ACTA2*** |  | low | 25449435 |
| ***SSTR2*** | Somatostatin Receptor 2 | low | 22640914 |
| ***SSTR5*** | Somatostatin Receptor 5 | low | 22640914 |
| ***STARD13*** | StAR-related lipid transfer domain 13 | low | 27844181 |
| ***STAT3*** | Signal Transducer And Activator Of Transcription 3 | high | 22977555 |
| ***STAT4*** |  | low | 24965572 |
| ***STMN1*** | Stathmin 1 | high | 22911364, 16739096 |
| ***STMN1, SPP1*** |  | joint expression | 16739096 |
| ***SYF2*** | SYF2 Pre-MRNA Splicing Factor | high | 26260052 |
| ***SYK*** | Spleen Associated Tyrosine Kinase | low | 17121887 |
| ***TFAP4*** | transcription Factor AP-4 | high | 23055200 |
| ***TGFB1*** | Transforming Growth Factor Beta 1 | low | 16270528 |
| ***THOC5*** | THO Complex 5 | high | 26549021 |
| ***THOP1*** | Thimet Oligopeptidase 1 | low | 24604581 |
| ***THY1*** | Thy-1 Cell Surface Antigen | high | 25271001, 21272924 |
| ***TJP1*** | Tight Junction Protein ZO-1 | low | 27750241 |
| ***TMSB10*** | thymosin beta 10 | high | 25037578 |
| ***TNFRSF12A*** | TNF Receptor Superfamily Member 12A | high | 23886137 |
| ***TNFSF10*** | Tumor Necrosis Factor Superfamily Member 10 | low | 25123818 |
| ***TNFSF11*** | Tumor Necrosis Factor Superfamily Member 11 | high | 17195907 |
| ***TNK2*** | Tyrosine Kinase Non Receptor 2 | high | 26536663, 25445114 |
| ***TREM1*** | Triggering Receptor Expressed On Myeloid Cells 1 | high | 25465376 |
| ***TRIM3*** | Tripartite Motif Containing 3 | low | 24994609 |
| ***TRIM44*** | Tripartite Motif Containing 44 | high | 27619678 |
| ***TTF1*** | RNA polymerase I | high | 26821084 |
| ***TWIST1*** | Twist Family BHLH Transcription Factor 1 | high | 27750241, 17987801 |
| ***TYMP*** | Thymidine Phosphorylase | high | 20442200 |
| ***UCA1*** | Urothelial Cancer Associated 1 (Non-Protein Coding) | high | 25760077 |
| ***ULBP1*** | UL16 Binding Protein 1 | low | 21756848 |
| ***UTS2*** | Urotensin 2 | high | 28105202 |
| ***VDAC1*** | Voltage Dependent Anion Channel 1 | high | 26831666 |
| ***VEGFA*** | Vascular Endothelial Growth Factor A | low | 21334407, 20442200 |
| ***VIL1*** | Villin-1 | high | 22530999 |
| ***VIM*** | Vimentin | high | 24833103 |
| ***WASF2*** | WAS Protein Family Member 2 | high | 17020969 |
| ***WIF1*** | WNT Inhibitory Factor 1 | low | 21052890 |
| ***WNT5A*** | Wnt Family Member 5A | low | 22493546 |
| ***WWP1*** | WW Domain Containing E3 Ubiquitin Protein Ligase 1 | high | 26506518 |
| ***XAF1*** | XIAP Associated Factor 1 | low | 18830757 |
| ***XPO4*** | Exportin 4 | low | 21332550 |
| ***ZNF148*** | Zinc Finger Protein 148 | low | 22372401 |
| ***ZYX*** | Zyxin | high | 16680155 |
