# Supplementary material for: Determining consistent prognostic biomarkers of overall survival and vascular invasion in hepatocellular carcinoma
Source: R Soc Open Sci. 2018 Dec 5;5(12):181006. doi: 10.1098/rsos.181006 (PMC6304123; doi:10.1098/rsos.181006)
Supplement: Supplementary Table 2 [file rsos181006supp2.docx]

**Determining consistent prognostic biomarkers of overall survival and vascular invasion in hepatocellular carcinoma**

Otília Menyhárt, Ádám Nagy, Balázs Győrffy

**Supplementary Table 2.**

**List of 226 biomarkers significant in the two ethnic groups including Cox proportional hazards regression *p*-values.** Red: 82 significant biomarker candidates were shared among Asian and White/Caucasian cohorts. Biomarkers significant only in the Asian cohort are labeled with green, and in the White/Caucasian cohort in blue. *p*-values in black are not significant.

| **Gene symbol** | **White cohort (*p*-value)** | **Asian cohort (*p*-value)** |
| --- | --- | --- |
| *ABCB1* | 0.026 | 0.015 |
| *ABCG2* | 0.56 | 0.027 |
| *ACE2* | 0.007 | 0.003 |
| *ACTA2* | 0.0011 | 0.078 |
| *ACVR1* | 0.12 | 0.063 |
| *ADAM10* | 0.083 | 0.26 |
| *ADH4* | 0.0031 | 0.0000018 |
| *ADRB2* | 0.11 | 0.000058 |
| *AFP* | 0.31 | 0.069 |
| *AFP* and *SPP1* | 0.12 | 0.029 |
| *AGER* | 0.0017 | 0.3 |
| *AIM2* | 0.17 | 0.29 |
| *AJAP1* | 0.43 | 0.061 |
| *AKAP12* | 0.052 | 0.1 |
| *AKT1* | 0.22 | 0.016 |
| *ALDH1A1* | 0.33 | 0.016 |
| *ALDH1L1* | 0.09 | 0.017 |
| *ALDH2* | 0.31 | 4.2E-09 |
| *ANGPT2* | 0.3 | 0.00000033 |
| *ANP32A* | 0.064 | 0.27 |
| *ANPEP* | 0.079 | 0.0017 |
| *ANXA4* | 0.048 | 0.24 |
| *APOD* | 0.012 | 0.0039 |
| *ARID1A* | 0.023 | 0.078 |
| *ARL6IP5* | 0.1 | 0.0019 |
| *ATF5* | 0.15 | 0.016 |
| *ATOH8* | 0.062 | 0.011 |
| *ATXN7* | 0.29 | 0.14 |
| *AURKA* | 0.22 | 0.00000096 |
| *BARX2* | 0.007 | 0.12 |
| *BATF2* | 0.092 | 0.3 |
| *BCL2L11* | 0.008 | 0.071 |
| *BCL3* | 0.086 | 0.022 |
| *BIRC3* | 0.055 | 0.24 |
| *BIRC5* | 0.037 | 1.9E-10 |
| *BLZF1* | 0.074 | 0.14 |
| *BMP7* | 0.011 | 0.0093 |
| *BTG1* | 0.0066 | 0.1 |
| *BTG3* | 0.1 | 0.1 |
| *BUB1B* | 0.042 | 0.000000024 |
| *CADM1* | 0.052 | 0.0004 |
| *CADM2* | 0.25 | 0.033 |
| *CAP2* | 0.035 | 0.019 |
| *CBS* | 0.18 | 0.000076 |
| *CBX4* | 0.027 | 0.33 |
| *CCNB1* | 0.018 | 6.3E-09 |
| *CCNF* | 0.041 | 0.000000071 |
| *CCR6* | 0.054 | 0.14 |
| *CCRL1* | 0.075 | 0.21 |
| *CD151* | 0.077 | 0.15 |
| *CD151* and *MET* | 0.012 | 0.2 |
| *CD24* | 0.025 | 0.024 |
| *CD274* | 0.11 | 0.34 |
| *CD274* and *CXCL12* | 0.019 | 0.074 |
| *CD276* | 0.23 | 0.0023 |
| *CD44* | 0.023 | 0.31 |
| *CDC20* | 0.016 | 2.5E-09 |
| *CDC25A* | 0.0091 | 0.00000067 |
| *CDC5L* | 0.037 | 0.19 |
| *CDCP1* | 0.26 | 0.07 |
| *CDH1* | 0.0083 | 0.031 |
| *CDH1* and *VIM* | 0.0083 | 0.04 |
| *CDK4* | 0.017 | 0.0000031 |
| *CDK5R2* | 0.0074 | 0.064 |
| *CDKN1B* | 0.24 | 0.31 |
| *CDKN1C* | 0.017 | 0.0054 |
| *CDKN1C* and *RHOA* | 0.047 | 0.000098 |
| *CDX1* | 0.092 | 0.0079 |
| *CENPH* | 0.064 | 0.000028 |
| *CHI3L1* | 0.12 | 0.012 |
| *CISD2* | 0.036 | 0.21 |
| *CKAP2* | 0.058 | 0.0000055 |
| *CKS1B* | 0.18 | 0.000026 |
| *CKS1B* and *CDKN1B* | 0.04 | 0.000074 |
| *CLDN7* | 0.18 | 0.00093 |
| *COL1A1* | 0.18 | 0.016 |
| *CPE* | 0.24 | 0.3 |
| *CTHRC1* | 0.11 | 0.00051 |
| *CTTN* | 0.086 | 0.23 |
| *CXCL12* | 0.027 | 0.06 |
| *CXCR4* | 0.082 | 0.039 |
| *CYTH2* | 0.18 | 0.027 |
| *DEK* | 0.093 | 0.016 |
| *DEPDC1* | 0.0022 | 0.00000026 |
| *DHX33* | 0.38 | 0.26 |
| *DIXDC1* | 0.23 | 0.057 |
| *DKC1* | 0.01 | 0.00069 |
| *DLX4* | 0.49 | 0.0032 |
| *DPYSL3* | 0.15 | 0.062 |
| *DUOX1* | 0.21 | 0.25 |
| *E2F1* | 0.024 | 0.000014 |
| *E2F3* | 0.14 | 0.000023 |
| *ECM1* | 0.015 | 0.016 |
| *EDIL3* | 0.14 | 0.035 |
| *EFEMP1* | 0.21 | 0.023 |
| *EIF3H* | 0.074 | 0.011 |
| *EIF4EBP1* | 0.071 | 0.47 |
| *ELAVL1* | 0.049 | 0.02 |
| *ENAH* | 0.063 | 0.0093 |
| *EP300* | 0.17 | 0.21 |
| *EPAS1* | 0.001 | 0.0035 |
| *EPCAM* | 0.0057 | 0.016 |
| *EPHA2* | 0.31 | 0.051 |
| *EPOR* | 0.22 | 0.018 |
| *ERBB2* | 0.024 | 0.024 |
| *EYA4* | 0.17 | 0.29 |
| *EZH2* | 0.018 | 0.00000035 |
| *FABP1* | 0.044 | 0.064 |
| *FAM83D* | 0.0014 | 0.000028 |
| *FBLN5* | 0.0046 | 0.00046 |
| *FERMT1* | 0.0071 | 0.23 |
| *FERMT2* | 0.18 | 0.00074 |
| *FLT4* | 0.001 | 0.0015 |
| *FOXD3* | 0.17 | 0.22 |
| *FOXF2* | 0.0037 | 0.0021 |
| *FOXK2* | 0.4 | 0.000000015 |
| *FOXM1* | 0.0036 | 0.0000014 |
| *FOXP1* | 0.029 | 0.0057 |
| *GABARAPL1* | 0.019 | 0.00015 |
| *GABPA* | 0.069 | 0.2 |
| *GADD45G* | 0.011 | 0.0016 |
| *GJA1* | 0.067 | 0.14 |
| *GLI1* | 0.3 | 0.15 |
| *GLTSCR2* | 0.23 | 0.021 |
| *GMNN* | 0.38 | 0.024 |
| *GOLM1* | 0.12 | 0.033 |
| *GOLPH3* | 0.19 | 0.036 |
| *GPC3* | 0.07 | 0.19 |
| *GPRC5A* | 0.2 | 0.13 |
| *HACE1* | 0.25 | 0.0019 |
| *HDGF* | 0.0003 | 0.0056 |
| *HIF1A* | 0.15 | 0.01 |
| *HIF1A* and *EPAS1* | 0.00078 | 0.0035 |
| *HINT2* | 0.13 | 0.0015 |
| *HLX* | 0.26 | 0.000034 |
| *HMGA1* | 0.0025 | 0.000021 |
| *HMGB1* | 0.078 | 0.27 |
| *HOTAIR* | 0.051 | 0.26 |
| *HOXB7* | 0.066 | 0.02 |
| *HPSE* | 0.42 | 0.019 |
| *HSPA2* | 0.33 | 0.16 |
| *ICAM1* | 0.36 | 0.061 |
| *ID1* | 0.012 | 0.33 |
| *ID2* | 0.021 | 0.042 |
| *IGF2BP3* | 0.052 | 0.00032 |
| *IGFBP1* | 0.072 | 0.074 |
| *IKBKG* | 0.2 | 0.075 |
| *IKZF1* | 0.014 | 0.062 |
| *IL18R1* | 0.012 | 0.04 |
| *IL2* | 0.066 | 0.17 |
| *IL8* | 0.1 | 0.00061 |
| *IMP3* | 0.3 | 0.0009 |
| *ING3* | 0.071 | 0.11 |
| *ING4* | 0.02 | 0.078 |
| *INPPL1* | 0.34 | 0.0021 |
| *IQGAP1* | 0.089 | 0.14 |
| *IQGAP2* | 0.15 | 0.0000073 |
| *KAL1* | 0.16 | 0.12 |
| *KDR* | 0.0052 | 0.00016 |
| *KIAA0114* | 0.038 | 0.0021 |
| *KIAA1524* | 0.05 | 0.00000004 |
| *KIF18A* | 0.014 | 0.000000019 |
| *KIF1B* | 0.15 | 0.2 |
| *KISS1* and *KISS1R* | 0.031 | 0.13 |
| *KIT* | 0.047 | 0.000025 |
| *KRT19* | 0.0031 | 0.02 |
| *L1CAM* | 0.094 | 0.072 |
| *LARP1* | 0.3 | 0.011 |
| *LIN28A* | 0.085 | 0.16 |
| *LOXL4* | 0.16 | 0.052 |
| *LYVE1* | 0.27 | 0.27 |
| *MACC1* | 0.17 | 0.0033 |
| *MAD2L1* | 0.045 | 0.0000026 |
| *MAGED1* | 0.019 | 0.17 |
| *MAGED4* | 0.39 | 0.015 |
| *MAGI1* | 0.21 | 0.000044 |
| *MAT1A* | 0.16 | 0.00012 |
| *MCAM* | 0.012 | 0.022 |
| *MELK* | 0.018 | 0.000000031 |
| *MEP1A* | 0.32 | 0.019 |
| *MET* | 0.043 | 0.0013 |
| *MFN2* | 0.18 | 0.036 |
| *MKI67* | 0.064 | 0.0000022 |
| *MMP12* | 0.11 | 0.0064 |
| *MMP7* | 0.11 | 0.00031 |
| *MTOR* | 0.26 | 0.053 |
| *NAT10* | 0.000044 | 0.00016 |
| *NCL* | 0.00039 | 0.011 |
| *NDRG1* | 0.02 | 0.004 |
| *NEDD9* | 0.032 | 0.095 |
| *NES* | 0.18 | 0.33 |
| *NEU1* | 0.0085 | 0.044 |
| *NKD1* | 0.086 | 0.086 |
| *NKX2-8* | 0.11 | 0.058 |
| *NNMT* | 0.0076 | 0.1 |
| *NODAL* | 0.096 | 0.21 |
| *NOTCH3* | 0.0038 | 0.35 |
| *NPAS2* | 0.06 | 0.0061 |
| *NPRL2* | 0.1 | 0.064 |
| *NRP1* | 0.09 | 0.12 |
| *NUAK1* | 0.2 | 0.28 |
| *PARK7* | 0.16 | 0.19 |
| *PDCD4* | 0.04 | 0.24 |
| *PDCD5* | 0.14 | 0.27 |
| *PDGFRA* | 0.094 | 0.046 |
| *PDK1* | 0.053 | 0.38 |
| *PDSS2* | 0.11 | 0.0099 |
| *PEBP1* | 0.302 | 0.00031 |
| *PECAM1* | 0.0095 | 0.31 |
| *PEMT* | 0.084 | 0.0001 |
| *PI4KA* | 0.027 | 0.1 |
| *PIWIL1* | 0.035 | 0.26 |
| *PKM2* | 0.22 | 0.0000001 |
| *PLAT* | 0.0012 | 0.014 |
| *PLAU* | 0.16 | 0.054 |
| *PLAU, PLAUR,* and *SERPINE1* | 0.55 | 0.052 |
| *PLAUR* | 0.21 | 0.0015 |
| *PLCE1* | 0.078 | 0.096 |
| *PLK1* | 0.034 | 0.000000003 |
| *PNKD* | 0.12 | 0.28 |
| *PNLIPRP3* | 0.014 | 0.39 |
| *POU5F1* | 0.091 | 0.11 |
| *PREX2* | 0.001 | 0.24 |
| *PRKCA* | 0.17 | 0.066 |
| *PRKDC* | 0.0096 | 0.0041 |
| *PRMT5* | 0.0044 | 0.031 |
| *PROM1* | 0.036 | 0.031 |
| *PRRX1* | 0.002 | 0.053 |
| *PTCH1* | 0.3 | 0.057 |
| *PTEN* | 0.22 | 0.000031 |
| *PTGS2* | 0.096 | 0.14 |
| *PTK2* | 0.15 | 0.026 |
| *PTOV1* | 0.045 | 0.11 |
| *PTP4A3* | 0.18 | 0.0028 |
| *PTTG1* | 0.026 | 0.000000052 |
| *PVRL4* | 0.35 | 0.18 |
| *PYGO2* | 0.0022 | 0.0011 |
| *RASSF10* | 0.013 | 0.11 |
| *RASSF5* | 0.093 | 0.0012 |
| *RCHY1* | 0.05 | 0.027 |
| *REG3A* | 0.067 | 0.25 |
| *REG3A* and *REG1A* | 0.15 | 0.026 |
| *RELN* | 0.14 | 0.15 |
| *RHOA* | 0.059 | 0.011 |
| *RHOC* | 0.14 | 0.065 |
| *RIPK1* | 0.078 | 0.058 |
| *ROR2* | 0.15 | 0.16 |
| *RORA* | 0.021 | 0.0012 |
| *RPS19BP1* | 0.009 | 0.16 |
| *RRAD* | 0.29 | 0.11 |
| *RRM2* | 0.045 | 0.000016 |
| *RUNX2* | 0.037 | 0.063 |
| *S100A14* | 0.15 | 0.18 |
| *SAMSN1* | 0.095 | 0.39 |
| *SEC62* | 0.41 | 0.0000011 |
| *SERPINE1* | 0.34 | 0.04 |
| *SFRP1* | 0.0064 | 0.37 |
| *SIRT1* | 0.053 | 0.063 |
| *SKP2* | 0.0081 | 0.019 |
| *SLC22A1* | 0.013 | 0.00000084 |
| *SLC2A1* | 0.0034 | 0.0001 |
| *SLC39A6* | 0.068 | 0.0098 |
| *SLC5A8* | 0.027 | 0.14 |
| *SLC7A11* | 0.00072 | 0.00031 |
| *SLC7A5* | 0.13 | 0.076 |
| *SLC9A1* | 0.06 | 0.013 |
| *SMARCA2* | 0.0057 | 0.026 |
| *SMARCB1* | 0.0027 | 0.21 |
| *SOCS2* | 0.000013 | 0.0000077 |
| *SOCS3* | 0.055 | 0.16 |
| *SOX1* | 0.086 | 0.098 |
| *SPC24* | 0.028 | 0.0025 |
| *SPHK1* | 0.47 | 0.00025 |
| *SPIB* | 0.055 | 0.14 |
| *SPP1* | 0.016 | 0.000057 |
| *SPP1* and *ACTA2* | 0.082 | 0.00044 |
| *SSTR2* | 0.29 | 0.017 |
| *SSTR5* | 0.23 | 0.34 |
| *STARD13* | 0.045 | 0.076 |
| *STAT3* | 0.0043 | 0.076 |
| *STAT4* | 0.0081 | 0.16 |
| *STMN1* | 0.022 | 0.00000042 |
| *STMN1* and *SPP1* | 0.0067 | 0.00000032 |
| *SYF2* | 0.2 | 0.034 |
| *SYK* | 0.11 | 0.22 |
| *TFAP4* | 0.0044 | 0.011 |
| *TGFB1* | 0.067 | 0.11 |
| *THOC5* | 0.00018 | 0.00099 |
| *THOP1* | 0.16 | 0.015 |
| *THY1* | 0.0063 | 0.052 |
| *THY1, CD24, PROM1* and *ANPEP* | 0.62 | 0.079 |
| *TJP1* | 0.026 | 0.0054 |
| *TMSB10* | 0.19 | 0.015 |
| *TNFRSF12A* | 0.011 | 0.17 |
| *TNFSF10* | 0.046 | 0.0099 |
| *TNFSF11* | 0.19 | 0.0072 |
| *TNK2* | 0.13 | 0.026 |
| *TREM1* | 0.079 | 0.0035 |
| *TRIM3* | 0.32 | 0.13 |
| *TRIM44* | 0.41 | 0.095 |
| *TTF1* | 0.39 | 0.0024 |
| *TWIST1* | 0.19 | 0.038 |
| *TYMP* | 0.05 | 0.037 |
| *UCA1* | 0.35 | 0.0048 |
| *ULBP1* | 0.014 | 0.003 |
| *UTS2* | 0.0044 | 0.075 |
| *VDAC1* | 0.37 | 0.0036 |
| *VEGFA* | 0.048 | 0.0071 |
| *VIL1* | 0.13 | 0.27 |
| *VIM* | 0.021 | 0.37 |
| *WASF2* | 0.0057 | 0.00012 |
| *WIF1* | 0.023 | 0.0054 |
| *WNT5A* | 0.39 | 0.28 |
| *WWP1* | 0.027 | 0.041 |
| *XAF1* | 0.2 | 0.00012 |
| *XPO4* | 0.22 | 0.021 |
| *ZNF148* | 0.17 | 0.097 |
| *ZYX* | 0.16 | 0.1 |
