# Supplementary material for: Determining consistent prognostic biomarkers of overall survival and vascular invasion in hepatocellular carcinoma
Source: R Soc Open Sci. 2018 Dec 5;5(12):181006. doi: 10.1098/rsos.181006 (PMC6304123; doi:10.1098/rsos.181006)
Supplement: Supplementary Table 3 [file rsos181006supp3.docx]

**Determining consistent prognostic biomarkers of overall survival and vascular invasion in hepatocellular carcinoma**

Otília Menyhárt, Ádám Nagy, Balázs Győrffy

**Supplementary Table 3.**

**Functional grouping of significant prognostic biomarker candidates in the Asian cohort by DAVID gene ontology analysis.**

| **Function** | ***p*-value** | **Genes included** | **FDR** |
| --- | --- | --- | --- |
| Cell division | 1.5E-10 | *CCNB1, FAM83D, CKS1B, MAD2L1, CCNF, BUB1B, BIRC5, AURKA, CDC20, PTTG1, CDK4, CDC25A* | 2.1E-07 |
| Anaphase-promoting complex-dependent catabolic process/cyclin degradation | 1.9E-08 | *CCNB1, MAD2L1, PLK1, BUB1B, AURKA, CDC20, PTTG1* | 2.6E-05 |
| Mitotic nuclear division | 5.4E-08 | *FAM83D, PLK1, CCNF, BUB1B, BIRC5, AURKA, CDC20, PTTG1, CDC25A* | 7.7E-05 |
| Sister chromatid cohesion | 9.2E-08 | *MAD2L1, PLK1, KIF18A, BUB1B, BIRC5, CDC20, CENPH* | 1.3E-04 |
| G2/M transition of mitotic cell cycle | 5.0E-07 | *CCNB1, PLK1, FOXM1, BIRC5, AURKA, CDC25A, MELK* | 7.1E-04 |
| Protein ubiquitination involved in ubiquitin-dependent protein catabolic process | 9.5E-07 | *CCNB1, MAD2L1, PLK1, BUB1B, AURKA, CDC20, PTTG1* | 1.4E-03 |
| Regulation of cell cycle | 7.3E-06 | *CCNB1, PLK1, FOXM1, CCNF, PTEN, CDC25A* | 1.0E-02 |
| Cell proliferation | 1.3E-05 | *FAM83D, CKS1B, MKI67, PLK1, BUB1B, PTEN, CDC25A, MELK* | 1.8E-02 |
| Positive regulation of ubiquitin-protein ligase activity involved in regulation of mitotic cell cycle transition | 2.3E-05 | *CCNB1, MAD2L1, PLK1, BUB1B, CDC20* | 3.3E-02 |
