# Supplementary material for: Determining consistent prognostic biomarkers of overall survival and vascular invasion in hepatocellular carcinoma
Source: R Soc Open Sci. 2018 Dec 5;5(12):181006. doi: 10.1098/rsos.181006 (PMC6304123; doi:10.1098/rsos.181006)
Supplement: Supplementary Table 4 [file rsos181006supp4.docx]

**Determining consistent prognostic biomarkers of overall survival and vascular invasion in hepatocellular carcinoma**

Otília Menyhárt, Ádám Nagy, Balázs Győrffy

**Supplementary Table 4**.

**List of 40 biomarker candidates associated with overall survival in the entire dataset including both Asian and White/Caucasian subjects at 0.1% FDR.** Bold faced genes are significantly associated with OS in a multivariate Cox regression.

| **Symbol** | **Gene name** | **Overall survival HR (95% CI), *p*** | **Worse prognosis (expression low-high)** | **References (PMID)** |
| --- | --- | --- | --- | --- |
| *SOCS2* | Suppressor Of Cytokine Signaling 2 | HR=0.35 (0.25-0.5) *p*=1.8E-9 | low | 27465557 |
| *SLC5A8* | Solute Carrier Family 5 Member 8 | HR=0.36 (0.26-0.52) *p*=2.7E-9 | low | 27465549 |
| *IL2* | Interleukin 2 | HR=0.38 (0.27-0.54) *p*=1.1E-8 | low | 20940284 |
| *SOX1* | Sex-determining region Y (SRY)-box 1 | HR=0.38 (0.27-0.54) *p*=1.3E-8 | low | 26191244 |
| *HOTAIR* | HOX Transcript Antisense RNA | HR=0.38 (0.27-0.54) *p*=1.6E-8 | low | 21327457 |
| *FOXD3* | Forkhead box D3 | HR=0.39 (0.28-0.56) *p*=5.1E-8 | low | 26112097 |
| *PNLIPRP3* | Pancreatic Lipase Related Protein 3 | HR=0.4 (0.28-0.56) *p*=5.3E-8 | low | 19640199 |
| *NKX2-8* | NK2 Homeobox 8 | HR=0.41 (0.29-0.58) *p*=2.2E-7 | low | 24678995 |
| *CDC20* | Cell Division Cycle 20 | HR=2.49 (1.72-3.59) *p*=5.1E-7 | high | 28246274 |
| *SLC7A11* | Solute Carrier Family 7 Member 11 | HR=2.41 (1.69-3.44) *p*=5.3E-7 | high | 23229496 |
| *BIRC5* | Baculoviral IAP Repeat Containing 5 | HR=2.34 (1.65-3.3) *p*=7.4E-7 | high | 17559540, 15547736, 12374680 |
| *SLC22A1* | Solute Carrier Family 22 Member 1 | HR=0.43 (0.3-0.61) *p*=9.2E-7 | low | 22439694 |
| *STMN1 and SPP1* | Stathmin 1 and Secreted Phosphoprotein 1 (Osteopontin) | HR=2.45 (1.69-3.57) *p*=1.2E-6 | high | 16739096 |
| *ALDH2* | Aldehyde dehydrogenase-2 | HR=0.42 (0.29-0.6) *p*=1.3E-6 | low | 28027570 |
| *STMN1* | Stathmin 1 | HR=2.24 (1.59-3.17) *p*=2.8E-6 | high | 22911364, 16739096 |
| *PLK1* | Polo Like Kinase 1 | HR=2.23 (1.58-3.15) *p*=3.2E-6 | high | 19725153 |
| *RASSF10* | Ras-association domain family 10 | HR=0.45 (0.32-0.63) *p*=3.3E-6 | low | 26701853 |
| ***SPP1*** | **Secreted Phosphoprotein 1 (Osteopontin)** | **HR=2.27 (1.59-3.23) *p*=3.5E-6** | **high** | **25449435, 17161983, 16739096, 15754002** |
| ***SLC2A1*** | **Solute Carrier Family 2 Member 1** | **HR=2.25 (1.58-3.19) *p*=3.7E-6** | **high** | **21334407** |
| *ADH4* | Alcohol Dehydrogenase 4 (Class II), Pi Polypeptide | HR=0.37 (0.24-0.58) *p*=4.3E-6 | low | 22147505 |
| *LIN28A* | Lin-28 Homolog A | HR=0.46 (0.32-0.64) *p*=5.2E-6 | low | 22429493 |
| ***EZH2*** | **Enhancer Of Zeste 2 Polycomb Repressive Complex 2 Subunit** | **HR=2.23 (1.56-3.19) *p*=6.8E-6** | **high** | **24966962** |
| *KIAA1524* | Cancerous Inhibitor Of Protein Phosphatase 2A | HR=2.19 (1.54-3.13) *p*=9E-6 | high | 22847158 |
| *PIWIL1* | Piwi Like RNA-Mediated Gene Silencing 1 | HR=0.46 (0.33-0.66) *p*=9.7E-6 | low | 21989785 |
| *PTTG1* | Pituitary Tumor-Transforming 1 | HR=2.14 (1.51-3.02) *p*=1.1E-5 | high | 16628605 |
| ***CCNF*** | **Cyclin F** | **HR=2.15 (1.51-3.05) *p*=1.2E-5** | **high** | **23305207** |
| *MAD2L1* | Mitotic Arrest Deficient 2 Like 1 | HR=2.25 (1.54-3.28) *p*=1.7E-5 | high | 18715617 |
| *KDR* | Kinase Insert Domain Receptor | HR=0.45 (0.31-0.66) *p*=2E-5 | low | 21270061 |
| ***KIF18A*** | **Kinesin Family Member 18A** | **HR=2.09 (1.47-2.95) *p*=2.2E-5** | **high** | **25431949** |
| *TREM1* | Triggering Receptor Expressed On Myeloid Cells 1 | HR=2.13 (1.49-3.06) *p*=2.4E-5 | high | 25465376 |
| *IQGAP2* | IQ Motif Containing GTPase Activating Protein 2 | HR=0.48 (0.34-0.68) *p*=2.5E-5 | low | 24998570 |
| ***WASF2*** | **WAS Protein Family Member 2** | **HR=2.1 (1.48-2.99) *p*=2.5E-5** | **high** | **17020969** |
| ***DEPDC1*** | **DEP Domain Containing 1** | **HR=2.08 (1.47-2.95) *p*=2.6E-5** | **high** | **25605201** |
| *HMGA1* | High Mobility Group AT-Hook 1 | HR=2.08 (1.47-2.95) *p*=2.6E-5 | high | 27855356 |
| *THOC5* | THO Complex 5 | HR=2.07 (1.46-2.93) *p*=2.9E-5 | high | 26549021 |
| *CDK4* | Cyclin Dependent Kinase 4 | HR=2.15 (1.49-3.1) *p*=3E-5 | high | 23292829 |
| *CCNB1* | Cyclin B1 | HR=2.34 (1.55-3.54) *p*=3.4E-5 | high | 22682366 |
| *FBLN5* | Fibulin-5 | HR=0.47 (0.33-0.68) *p*=3.6E-5 | low | 25494879 |
| *MELK* | Maternal Embryonic Leucine Zipper Kinase | HR=2.22 (1.5-3.27) *p*=3.7E-5 | high | 27798878 |
| ***PKM2*** | **Pyruvate kinase M2** | **HR=2.07 (1.45-2.95) *p*=3.9E-5** | **high** | **25788265, 24466275** |

Statistical test: Cox univariate regression analysis. HR: hazard rate. CI: confidence interval.
