# Supplementary material for: Determining consistent prognostic biomarkers of overall survival and vascular invasion in hepatocellular carcinoma
Source: R Soc Open Sci. 2018 Dec 5;5(12):181006. doi: 10.1098/rsos.181006 (PMC6304123; doi:10.1098/rsos.181006)
Supplement: Supplementary Table 5 [file rsos181006supp5.docx]

**Determining consistent prognostic biomarkers of overall survival and vascular invasion in hepatocellular carcinoma**

Otília Menyhárt, Ádám Nagy, Balázs Győrffy

**Supplementary Table 5**.

**List of 52 biomarker candidates associated with vascular invasion based on literature search.**

| **Symbol** | **GENE Name** | **PMID** |
| --- | --- | --- |
| ***ADRB2*** | Adrenoceptor Beta 2 | 22588469 |
| ***AFP*** | Alpha Fetoprotein | 15305374 |
| ***AIM2*** | Absent In Melanoma 2 | 28580773 |
| ***AJAP1*** | Adherens Junctions Associated Protein 1 | 26122373 |
| ***ANP32A*** | Acidic Nuclear Phosphoprotein 32 Family Member A | 20683644 |
| ***APOA1*** | Apolipoprotein A1 | 21649721 |
| ***CADM2*** | Cell Adhesion Molecule 2 | 24240726 |
| ***CD151*** | CD151 Molecule (Raph Blood Group) | 19065669 |
| ***CYTH2*** | Cytohesin 2 | 23545718 |
| ***ECM1*** | Extracellular Matrix Protein 1 | 21128013 |
| ***EDIL3*** | EGF Like Repeats And Discoidin Domains 3 | 25273699 |
| ***ENG*** | Endoglin | 25286761 |
| ***FABP1*** | Fatty Acid Binding Protein 1 | 25436304 |
| ***FAM83D*** | Family With Sequence Similarity 83 Member D | 26125229 |
| ***FLT4*** | Fms Related Tyrosine Kinase 4 | 12017318 |
| ***FOXK2*** | Forkhead Box K2 | 28506857 |
| ***GPC3*** | Glypican-3 | 22654434 |
| ***GPRC5A*** | G Protein-Coupled Receptor Class C Group 5 Member A | 23632812 |
| ***HACE1*** | HECT domain and ankyrin repeat containing E3 ubiquitin protein ligase 1 | 27805249 |
| ***HIF1A*** | Hypoxia Inducible Factor 1 Alpha Subunit | 24374892, 19948069 |
| ***HLX*** | H2.0-like homeobox 1 | 26631039 |
| ***ICAM1*** | Intercellular Adhesion Molecule 1 | 26667486 |
| ***ID2*** | Inhibitor Of DNA Binding 2, HLH Protein | 18281534 |
| ***KAL1*** | Kallmann syndrome-1, Anosmin 1 | 25892360 |
| ***KIF18A*** | Kinesin Family Member 18A | 25431949 |
| ***MAGED4*** | MAGE Family Member D4 | 24068544 |
| ***MET*** | MET Proto-Oncogene, Receptor Tyrosine Kinase | 19065669 |
| ***NAA10*** | N(Alpha)-Acetyltransferase 10, NatA Catalytic Subunit | 23258102 |
| ***NDRG1*** | N-Myc Downstream Regulated 1 | 17170744 |
| ***NEDD9*** | Neural Precursor Cell Expressed, Developmentally Down-Regulated 9 | 25812772 |
| ***NODAL*** | Nodal Growth Differentiation Factor | 24465741 |
| ***NRP1*** | Neuropilin1 | 26563279 |
| ***PDGFRA*** | Platelet Derived Growth Factor Receptor Alpha | 25333264 |
| ***PDIA6*** | Protein Disulfide Isomerase Family A Member 6 | 21649721 |
| ***PEBP1*** | Phosphatidylethanolamine Binding Protein 1 | 20739083 |
| ***PECAM1*** | Platelet And Endothelial Cell Adhesion Molecule 1 | 12017318 |
| ***PLCE1*** | Phospholipase C Epsilon 1 | 28031722 |
| ***PTP4A3*** | Protein Tyrosine Phosphatase Type IVA, Member 3 | 23064776 |
| ***PVRL4*** | Nectin Cell Adhesion Molecule 4 | 26793002 |
| ***PYGO2*** | Pygopus Family PHD Finger 2 | 25545771 |
| ***RASSF5*** | Ras Association Domain Family Member 5 | 24563371 |
| ***REG3A*** | Regenerating Family Member 3 Alpha | 15814635 |
| ***RORA*** | RAR Related Orphan Receptor A | 24798975 |
| ***RPS19BP1*** | Ribosomal Protein S19 Binding Protein 1 | 26339164 |
| ***S100A14*** | S100 Calcium Binding Protein A14 | 23886191 |
| ***SFRP1*** | Secreted frizzled-related protein-1 | 26851021 |
| ***STAT4*** | Signal Transducer And Activator Of Transcription 4 | 24965572 |
| ***STMN1*** | Stathmin 1 | 22911364 |
| ***TRIM44*** | Tripartite Motif Containing 44 | 27619678 |
| ***VEGFB*** | Vascular Endothelial Growth Factor B | 18537151 |
| ***VIL1*** | Villin-1 | 22530999 |
| ***XAF1*** | XIAP Associated Factor 1 | 18830757 |
